# Polyoxygenated Steroids from the Octocoral *Leptogorgia punicea* and *in Vitro* Evaluation of Their Cytotoxic Activity

**DOI:** 10.3390/md12125864

**Published:** 2014-12-04

**Authors:** Maria Izabel G. Moritz, Lucas Lourenço Marostica, Éverson M. Bianco, Maria Tereza R. Almeida, João L. Carraro, Gabriela M. Cabrera, Jorge A. Palermo, Cláudia M. O. Simões, Eloir P. Schenkel

**Affiliations:** 1 Post-Graduation Program in Pharmacy, Federal University of Santa Catarina, Florianópolis, SC 88040-900, Brazil; E-Mails: mizabelgm@gmail.com (M.I.G.M.); lucasmarostica@yahoo.com.br (L.L.M.); ebianco@chemist.com (E.M.B.); terezarojo@gmail.com (M.T.R.A.); claudia.simoes@ufsc.br (C.M.O.S.); 2 Invertebrates Department, National Museum, Federal University of Rio de Janeiro, Rio de Janeiro 20940-040, RJ, Brazil; E-Mail: joao.porifera@gmail.com; 3 UMYMFOR—Department of Organic Chemistry, FCEN, University of Buenos Aires, Buenos Aires C1428EGA, Argentina; E-Mails: palermo@qo.fcen.uba.ar (J.A.P.); gabyc@qo.fcen.uba.ar (G.C.)

**Keywords:** *Leptogorgia punicea*, octocoral, polyoxygenated steroids, cytotoxic effects, A549 cells, lung cancer cells

## Abstract

Five new polyoxygenated marine steroids—punicinols A–E (**1**–**5**)—were isolated from the gorgonian *Leptogorgia punicea* and characterized by spectroscopic methods (IR, MS, ^1^H, ^13^C and 2-D NMR). The five compounds induced *in vitro* cytotoxic effects against lung cancer A549 cells, while punicinols A and B were the most active, with IC_50_ values of 9.7 μM and 9.6 μM, respectively. The synergistic effects of these compounds with paclitaxel, as well as their effects on cell cycle distribution and their performance in the clonogenic assay, were also evaluated. Both compounds demonstrated significant synergistic effects with paclitaxel.

## 1. Introduction

Gorgonian octocorals are widely distributed marine organisms, and have provided a wide range of new compounds with diverse carbon skeletons and valuable biological activities [1,2]. *Leptogorgia punicea* (Octocorallia, Gorgonaea) is a reddish purple species, which is found in Brazil, from the coast of Santa Catarina State in the South to the State of Maranhão in the North [3].

The genus *Leptogorgia* Milne Edwards & Haime, 1857, (Gorgoniidae) comprises approximately 54 valid species and has been found in the Atlantic Ocean, the Caribbean and Mediterranean seas, around Southern Africa, and in the Subantarctic regions [4]. Different groups of substances, such as cembrane diterpenoids [5,6,7,8,9,10,11], prenylated alkaloids [12] and polyoxygenated steroids [13,14,15,16] were isolated from *Leptogorgia* genus (=*Lophogorgia*). There have been few reports specifically on *Leptogorgia punicea*; Epifanio described the isolation of an uncommon polyoxygenated sterol, punicin (6β-acetoxycholestane-3β,5α,17α-triol), and a benzohydroquinone from *Lophogorgia punicea* (=*Leptogorgia punicea*), collected in the State of Rio de Janeiro, Brazil [15].

Marine steroids have shown promising activity against tumor cells, although steroids are currently used as anti-inflammatory, hormonal and contraceptive drugs [17,18,19]. One of the most powerful cell growth inhibitors, the steroid dimer cephalostatin 1, was isolated from the invertebrate chordate *Cephalodiscus gilchristi* and has become a prototype for many other cephalostatins [19,20]. Other examples of active marine steroids, are the polyoxygenated steroids that can be found with high diversity in marine invertebrates, and that also show cytotoxic activity against several tumor cell lines, such as those isolated from the Mediterranean gorgonian *Leptogorgia sarmentosa* [16,21] and also those isolated from the Antarctic octocoral *Dasystenella acanthine*, which were cytotoxic against the LN-caP (breast adenocarcinoma) and K-562 (chronic myelogenous leukemia) cell lines [22].

A previous study of the extracts of *Leptogorgia punicea* led to the detection of antibiotic activity against *Staphylococcus aureus*, antifungal activity against *Candida albicans*, and anti-infective activity against *Trypanossoma cruzi* [23]. Furthermore, one of the fractions obtained from the ethanol extract displayed promising cytotoxic activity against A549 cells (human non-small cell lung carcinoma cells). The previous results, together with the abundance of the species in South Brazil, especially on the Santa Catarina coast, prompted us to investigate chemically and biologically *L. punicea* which led to five new cytotoxic polyoxygenated steroids namely punicinols A–E (**1**–**5**). To obtain additional pharmacological data about the most active compounds, their synergistic effects with paclitaxel, as well as their effects on cell cycle distribution and their performance in a clonogenic activity assay, were also evaluated.

## 2. Results and Discussion

### 2.1. Chemistry

The *n*-hexane fraction from the octocoral *Leptogorgia punicea* was fractionated by repeated column chromatography on silica gel (see experimental section). The most cytotoxic fraction was further purified by reversed-phase HPLC, resulting in the isolation of compounds **1**–**5**.

**Figure 1:**
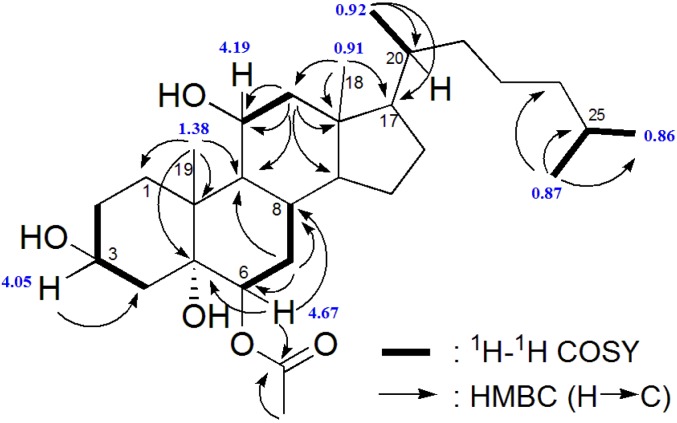
^1^H-^1^H COSY and HMBC correlations of compound **1**.

Compound **1** was isolated as a white powder. The molecular formula was established as C_29_H_50_O_5_, indicated by the cationized molecular ion [M + NH_4_]^+^ at *m/z* 496.3964 observed in the HR ESI-MS, corresponding to five degrees of unsaturation. The ^13^C NMR spectrum showed 29 carbon signals (Table 1), including four quaternary carbons, ten methylene, nine methine and six methyl groups, which were assigned by a HSQC/DEPT spectrum. The presence of an ester derivative was deduced by IR absorption bands 1714, 1259 and 1037 cm^−1^ and ^13^C-NMR (δ 170.3). Since the spectral data indicate only one signal of sp^2^ carbon, the remaining four double bond equivalents were attributed to a tetracyclic skeleton. This fact, together with the characteristic methyl groups at δ 0.86 (d), δ 0.87 (d), δ 0.92 (d), δ 0.91 (s) and at δ 1.38 (s) observed in the ^1^H NMR spectrum (Table 2), suggested the presence of a steroid structure. According to the MS2 spectrum obtained by a CID experiment on *m/z* 496 ([M + NH_4_]^+^), the peaks observed at *m/z* 478.3891, *m/z* 461.3619 and *m/z* 443.3518 were explained by successive H_2_O eliminations, suggesting the presence of a hydroxylated compound. The ^13^C NMR and ^1^H NMR spectra supported the presence of hydroxyl groups, namely one tertiary hydroxyl bound to the carbon at δ75.9 (C), and two secondary hydroxyl groups related to the carbinolic signals at δ_C_ 69.0 (CH)/δ_H_ 4.19 and δ_C_ 67.2 (CH)/δ_H_ 4.05, respectively. The presence of an additional oxygenated tertiary carbon at δ_C_ 75.7 (CH) bearing a deshielded hydrogen at δ 4.67 (dd, *J* = 3.2 Hz, appearing as an apparent triplet), suggested the point of esterification. This last carbon was identified as C-6, due to the HMBC correlations between δ 4.67 (H-6) and carbons at δ 170.3, δ 75.9 and δ 27.1 (Figure 1), attributed to C-5 and C-8, respectively. COSY correlations observed between the hydrogen at δ 1.70 (H-7) with both hydrogens at δ 4.67 (H-6) and δ 1.96 (H-8) confirmed the assignments in this part of ring B. The small coupling constants of the signal of H-6 (*J =* 3.2 Hz) with the hydrogens of H-7 indicated that the latter was equatorial, thus establishing a β-orientation for the acetate group.

The COSY spectrum revealed the coupling pattern in ring A. The large half-peak-width of H-3 (m, W_1/2_ = 19.6) are consistent with an axial position, establishing a β-orientation of the hydroxyl at C-3. Moreover, the HMBC correlations of the methyl singlet at δ 1.38 (Me-19) with the methylene at δ 31.8/C-1 and with other three carbons (C-5, C-9 and C-10) are consistent with ring A of the cholestane skeleton (Figure 1). An additional hydroxyl group was located at C-5 based on its ^13^C chemical shift δ75.9 (q) and on the HMBC correlations with H-6 and H-19.

The remaining hydroxyl group was placed at C-11, based on the HMBC correlations of the hydrogen at δ 4.19 with C-9 and C-12. The β-orientation of the hydroxyl attached on this position (C-11) was sustained by the coupling constants of the equatorial hydrogen at δ 4.19 (dd, 6.5, 3.2), with the *J* (ee) of 3.2 Hz [24,25].

**Table 1:** ^13^C NMR data of compounds **1**–**5** in CDCl_3_ (125 MHz; ppm).

Position	1	2	3	4	5
1	31.8	32.0	32.3	32.2	32.4
2	30.5	30.7	30.9	30.9	31.0
3	67.2	67.3	67.6	67.6	67.6
4	40.4	40.5	40.8	40.8	40.9
5	75.9	75.3	76.3	76.3	75.7
6	75.7	75.9	76.0	76.1	76.3
7	32.2	31.6	32.6	32.6	32.0
8	27.1	30.9	27.5	27.6	31.3
9	48.0	45.5	48.5	48.5	45.9
10	39.0	38.5	39.4	39.4	38.9
11	69.0	21.3	68.3	69.4	21.5
12	49.4	34.7	49.6	49.6	34.5
13	41.7	40.5	42.0	42.0	47.1
14	57.4	55.4	57.9	57.9	55.8
15	23.8	23.9	28.6	28.4	24.3
16	24.1	28.1	24.4	24.4	27.6
17	56.8	56.4	56.9	57.0	56.6
18	14.8	61.2	15.4	15.4	60.7
19	19.6	16.5	20.0	20.0	16.9
20	35.8	36.3	40.5	40.3	40.3
21	18.7	19.3	21.3	21.2	22.7
22	36.1	36.3	138.1	133.7	137.8
23	27.8	23.6	126.0	135.5	127.8
24	39.5	39.5	42.3	-	42.3
25	28.0	28.0	28.9	31.3	28.9
26	22.5	22.6	22.7	23.2	22.7
27	22.8	22.8	22.7	23.2	22.7
6-OAc	170.3	171.3	170.7	170.7	170.5
	21.5	21.4	21.9	21.8	21.8

The elucidation of a typical cholestane side chain was completed by COSY and HMBC correlations. The proposed relative configuration of **1** was confirmed by analysis of the NOESY spectrum (NOESY H/H: 3α/2α, 3α/4α, 11α/1, 11α/7, 11α/12α, 11α/12β, 11α/9, 6α/7, 6α/4α, 18Me/16β, 18Me/15β, 18Me/19Me, 18Me/20, 18Me/CH_3_-COO, 19Me/1, 19Me/4β, 19/8β, 19Me/6α, CH_3_-COO, 21Me/CH_3_-COO, 21Me/12β). The relative configuration at C-11 was established by a diagnostic NOESY correlation between H-11 and H-1 equatorial, which is only possible if H-11 is also equatorial. As for the relative configuration of C-6, there was also a NOESY correlation between H-6 and H-4 equatorial. The identity of this last proton was confirmed by the NOESY correlation of H-4 axial with the Me-19. The NOESY correlation between Me-19 and H-4 axial is only possible with an A-B transfusion and consequently OH-5 must be α, which also explains the chemical shift of C-5 (δ 75.9). The compound (**1**) was then established as 6β-acetoxycholestane-3β,5α,11β-triol, for which the name punicinol A is proposed.

**Table 2:** ^1^H NMR data of compounds **1**–**5** in CDCl_3_ (500 MHz; J in Hz; δ in ppm).

Position	1	2	3	4	5
1	1.65 (m ^a^)	1.45 (m ^a^)	1.65 (m ^a^)	1.65 (m ^a^)	1.45 (m ^a^)
2α	1.87 (m ^a^)	1.86 (m ^a^)	1.87 (m ^a^)	1. 87 (m ^a^)	1.86 (m ^a^)
2β	1.58 (m ^a^)	1.52 (m ^a^)	1.57 (m ^a^)	1.58 (m ^a^)	1.54 (m ^a^)
3	4.05 (m ^a^)	4.09 (m ^a^)	4.05 (m ^a^)	4.06 (m ^a^)	4.08 (m ^a^)
4α	1.54 (m ^a^)	1.58 (m ^a^)	1.54(m ^a^)	1.56(m ^a^)	1.57 (m ^a^)
4β	1.95 (m ^a^)	1. 85 (m ^a^)	1.95 (m ^a^)	1.96 (m ^a^)	1.85 (m ^a^)
6	4.67 (dd, 3.2,3.2)	4.7 (dd, 3.0,3.0)	4.66 (dd, 3.0,3.0)	4.66 (dd, 3.1,3.1)	4.70 (dd, 2.8,2.8)
7	1.70 (m ^a^)	1.61 (m ^a^)	1.70 (m ^a^)	1.70 (m ^a^)	1.59 (m ^a^)
8	1.96 (m ^a^)	1.68 (m ^a^)	1.95 (m ^a^)	1.96 (m ^a^)	1.67 (m ^a^)
9	1.51 (m ^a^)	1.38 (m ^a^)	1.50 (m ^a^)	1.51 (m ^a^)	1.38 (m ^a^)
11α	4.19 (dd, 6.5, 3.2)	1.48 (m ^a^)	4.19 (dd, 6.5, 3.2)	4.19 (dd, 6.3, 3.3)	1.48 (m ^a^)
11β	-	1.31 (m ^a^)	-	-	1.30 (m ^a^)
12α	1.45 (m ^a^)	1.04 (m ^a^)	1.44 (m ^a^)	1.46 (m ^a^)	1.00 (m ^a^)
12β	2.15 (dd, 14.0, 2.7)	2,46 (td, 12.7, 3.0)	2.11 (dd, 14.0, 2.7)	2.11 (dd, 14.2, 2.9)	2.42 (td, 12.7, 3.0)
14	1.08 (m ^a^)	1.24 (m ^a^)	1.08 (m ^a^)	1.07 (m ^a^)	1.21 (m ^a^)
15α	1.32 (m ^a^)	1.57 (m ^a^)	1.66 (m ^a^)	1.64 (m ^a^)	1.55 (m ^a^)
15β	1.10 (m ^a^)	1.01 (m ^a^)	1.26 (m ^a^)	1.25 (m ^a^)	0.95 (m ^a^)
16α	1.59 (m ^a^)	1.89 (m ^a^)	1.55(m ^a^)	1.57 (m ^a^)	1.75 (m ^a^)
16β	1.08 (m ^a^)	1.41 (m ^a^)	1.08 (m ^a^)	1.08 (m ^a^)	1.42 (m ^a^)
17	1.06 (m ^a^)	1.20 (m ^a^)	1.10 (m ^a^)	1.08 (m ^a^)	1.28 (m ^a^)
18a	0.91 (s)	3.73 (dd, 11.4, 4.7)	0.93 (s)	0.92 (s)	3.62 (d, 11.7)
18b		3.61 (dd, 11.4, 4.7)			3.55 (d, 11.7)
19	1.38 (s)	1.15 (s)	1.38 (s)	1.38 (s)	1.15 (s)
20	1.36 (m ^a^)	1.57 (m ^a^)	2.01 (m ^a^)	1.97 (m ^a^)	2.29 (dd, 15.3, 8.4)
21	0.92 (d, 6.6)	1.03 (d, 6.6)	1.01 (d, 6.5)	1.00 (d, 6.6)	1.09 (d, 6.6)
22	1.32 (m ^a^) 0.98 (m ^a^)	1.37 (m ^a^) 1.04 (m ^a^)	5.16 (dd, 15.0, 9.0)	5.13 (dd, 15.0, 8.4)	5.33 (dd, 15.0, 8.5)
23	1.81 (m ^a^) 1.25 (m ^a^)	1.18 (m ^a^) 1,33 (m ^a^)	5.26 (dd, 15.0, 7.0)	5.26 (dd, 15.0, 6.6)	5.37 (dd, 15.0, 6.5)
24	1.11 (m ^a^)	1.13 (m ^a^)	1.82 (m ^a^)	-	1.85 (m ^a^)
25	1.51 (m ^a^)	1.51 (m ^a^)	1.56 (m ^a^)	2.18 (m ^a^)	1.58 (m ^a^)
26	0.86 (d, 6.6)	0.86 (d, 6.6)	0.86 (d, 6.7)	0.94 (d, 6.6)	0.87 (d, 6.7)
27	0.87 (d, 6.6)	0.87 (d, 6.6)	0.86(d, 6.7)	0.94 (d, 6.6)	0.87 (d, 6.7)
6-OAc	2.07 (s)	2.06 (s)	2.07(s)	2.07 (s)	2.06 (s)

^a^ Signals overlapped.

Compound **2** was isolated as a white powder. The molecular formula was found to be C_29_H_50_O_5_, as indicated by the cationized molecule [M + Na]^+^ at *m/z* 501.3540. It corresponds to five double bond equivalents. The ^13^C NMR and ^1^H NMR spectra were similar to those of compound 1, which has the same molecular formula, but with two different carbon resonances. Thus, compound 2 had four quaternary carbons, 12 methylenes, eight methines and five methyl groups and the difference was the presence of an oxidized methylene, with protons resonating at δ 3.73 (d, *J* = 11.4 Hz, H-18) and δ 3.61 (d, *J* = 11.4 Hz, H-18), bound to a carbon at δ 61.2. Furthermore, a typical methyl singlet corresponding to C-18 was not observed, neither the signal of an oxidized C-11. Analysis of the 2D NMR spectra confirmed that in compound **2**, C-18 was oxidized to an alcohol instead of C-11. The structure of compound **2** was then established as 6β-acetoxycholestane-3β,5α,18-triol, and named punicinol B.

Compound **3** was isolated as a white powder. A molecular formula C_29_H_48_O_5_ was indicated by the HR ESI MS which gave the cationized molecule [M + Na]^+^ at *m/z* 499.3406 and corresponded to six double bond equivalents. The NMR spectra were closely related to those of compound **1**, displaying identical signals for rings A, B, C and D (Table 1 and Table 2). The main difference observed was in the nature of the side chain. The ^13^C NMR, ^1^H NMR and HSQC/DEPT spectra established the presence of a disubstituted double bond in the side chain, showing two olefinic hydrogens at δ 5.16 (dd, *J* = 15.0, 9.0 Hz) and δ 5.26 (dd, *J* = 15.0, 7.0 Hz) attached to C-22 (δ 138.1) and C-23 (δ 126.0), respectively. The coupling constant of 15.0 Hz between H-22 and H-23 indicated an *E* configuration at C-22 and C-23 of the double bond. The HMBC between the hydrogen of the methyl at δ 1.01 (d, *J* = 6.5 Hz) and the carbon at δ 138.1 (C-22), together with the COSY between de hydrogen of the double bond at δ 5.26 and the hydrogen at δ 1.82 (position 24) confirm the location of the double bond.

NOESY correlations were similar to those of compound 1, which confirms the same relative configuration of C-3 and C-6. Thus, the new compound (**3**) was established as 6β-acetoxycholest-22-ene-3β,5α,11β-triol, and was named punicinol C.

**Figure 2:**
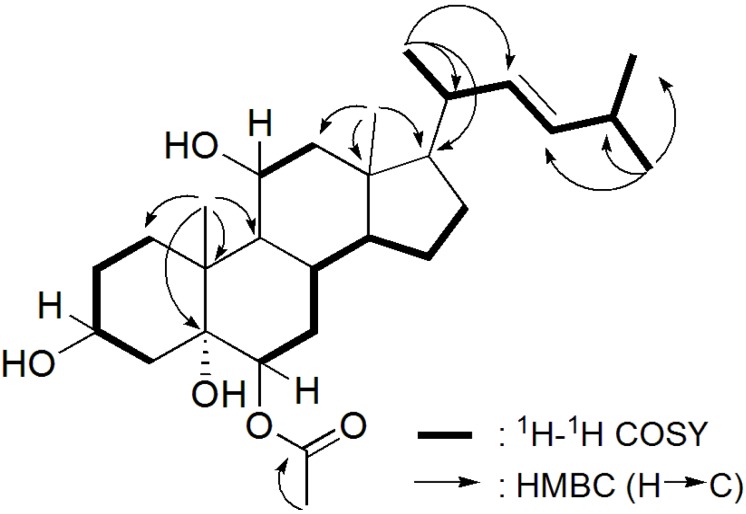
^1^H-^1^H COSY and HMBC Correlations of compound **4**.

Compound **4** was isolated as a white powder and the molecular formula was established as C_28_H_46_O_5_ by based on the cationized molecule at *m/z* 485.3261 ([M + Na]^+^) in the MS spectrum. In contrast with the other compounds, the ^13^C NMR spectrum showed 28 carbons (Table 1), which comprised four quaternary carbons, seven methylenes, 11 methines and six methyl groups. The ^1^H NMR spectrum also presented resonances corresponding to a double bond, at δ 5.13 (dd, *J* = 15.0, 8.4 Hz) and δ 5.26 (dd, *J* = 15.0, 6.6 Hz) (Table 2). Detailed comparison between compounds **3** and **4**, led to the conclusion that **4** had a shorter side chain, which was elucidated by COSY and HMBC correlations (Figure 2). The *E* configuration at C-22 and C-23 of the double bond was established by the coupling constant of 15.0 Hz between H-22 and H-23. Thus, the new compound (**4**) was established as 24-nor-6β-acetoxycholest-22-ene-3β,5α,11β-triol, and named punicinol D.

Compound **5** was isolated as a white powder. The molecular formula of C_29_H_48_O_5_ was determined from the cationized molecule [M + Na]^+^ at *m/z* 499.3394 observed in the HR ESI mass spectrum. The ^13^C NMR and ^1^H NMR spectra presented 29 carbons showing the same pattern of the steroid skeleton as was described for compound **2**, with an acetyloxy group located at C-6 and hydroxy groups located at C-3, C-5 and C-18 (Table 1). Same as compound **3**, compound **5** contains also a *E* substituted double bond in the side chain, with the olefinic hydrogens resonating at δ 5.33 (H-22, dd, *J* = 15.0, 8.5 Hz) and δ 5.37 (H-23, dd, *J* = 15.0, 6.5 Hz), attached to C-22 and C-23, respectively. Thus, compound **5** was defined as 6β-acetoxycholest-22-ene-3β,5α,18-triol, and was named punicinol E.

Punicin, a polyhydroxylated steroid previously isolated from *Leptogorgia punicea* collected in Rio de Janeiro, Brazil [15] presents the same steroid skeleton of punicinols A and B, but differs from the position of one of the hydroxyls. Similar cytotoxic polyoxygenated cholestanes were also isolated from other gorgonians such as *Sinularia* sp., *Isis hipuppuris* and *Sarcophyton elegans* [26,27,28]. To our knowledge, this is the first report of isolation and characterization of these five compounds.

### 2.2. Biological Activity

**Figure 3:**
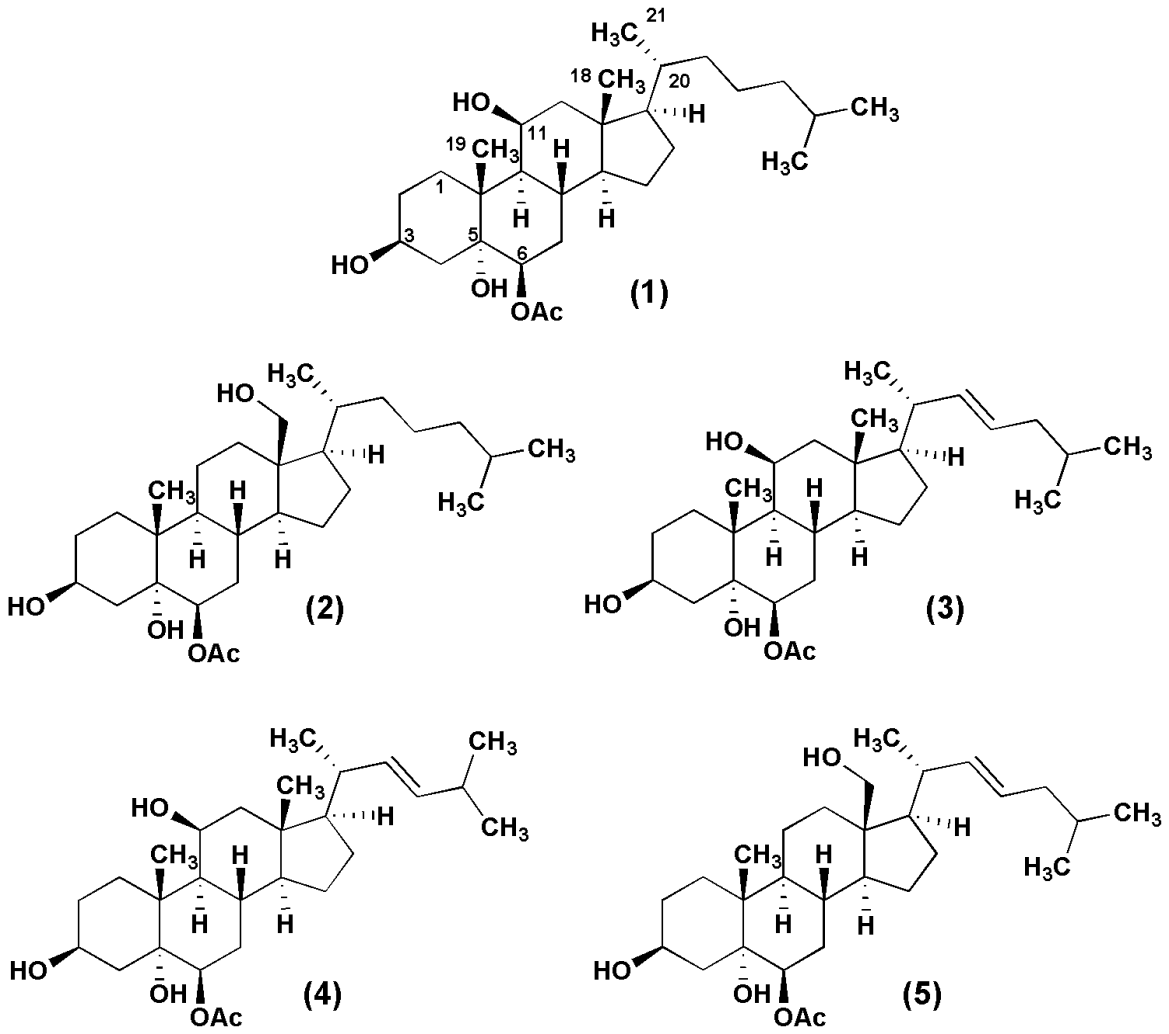
The chemical structures of **1**–**5**.

The ability of punicinols A–E (**1**–**5**) (Figure 3) to inhibit A549 cell proliferation was evaluated, after a preliminary test with the F1 fraction, which showed an IC_50_ of 5.13 μg/mL, Compounds **1**–**5** exhibited IC_50_ values ranging from 9.6–81.3 µM, depending on the length of treatment (24 or 48 h). Punicinols A and B were the most active compounds, even more active than cisplatin, which was used as positive control (Table 3). These compounds showed similar IC_50_ values to those obtained by Iwamaru and co-workers (2007), who reported the cytotoxic activity of new marine compounds isolated from gorgonian octocorals against malignant glioma cells [29]. Recently, Wang and co-workers (2013) described the cytotoxic effects of 14 new polyoxygenated steroids from the gorgonian *Menella kanisa.* These new steroids were active against lung (A549) and osteosarcoma (MG-63) cell lines [30].

**Figure 4:**
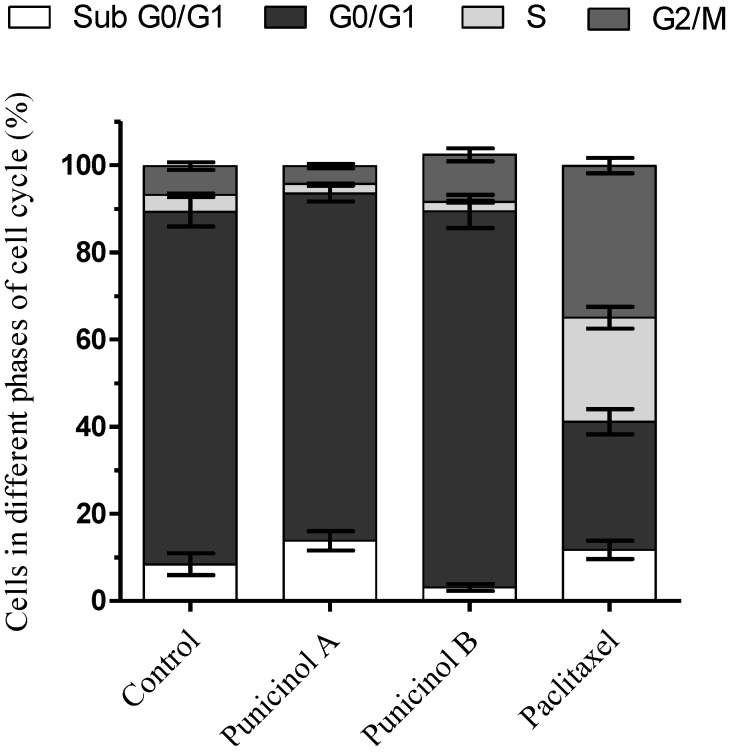
Effects of punicinols A (**1**) and B (**2**) isolated from *Leptogorgia punicea* on cell cycle distribution of A549 cells after 24 h of treatment. Data represent the mean ± standard deviations of cell population at different phases of cell cycle obtained with experiments in triplicate. Paclitaxel (0.25 µM) was used as positive control.

The effects of the most active compounds, punicinols A and B, were then evaluated on cell cycle distribution by flow cytometry. In comparison with the untreated controls, punicinol A increased the number of cells in the sub G0/G1 phase by 13.8%, while punicinol B increased the cells in the G2/M phase by 10.8% (Figure 4). These results show that punicinols A and B act at different stages of the cell cycle, and may partially explain the mechanism by which they inhibit cell proliferation and induce cytotoxicity to A549 cells. Iwamaru and colleagues (2007) also showed that compounds isolated from gorgonian octocorals modulate the cell cycle of glioma cells by inducing blockade of G2M phase [29].

**Table 3:** Cytotoxic *in vitro* activity against non-small cell lung cancer line (A549 cells) after 24 and 48 h of treatment. Values represent the mean ± standard deviations of three independent experiments.

Punicinol	IC_50_ 24 h (µM)	IC_50_ 48 h (µM)
A	11.8 ± 1.4	9.6 ± 1.1
B	13.6 ± 2.1	9.7 ± 1.7
C	47.9 ± 1.5	35.9 ± 1.4
D	81.3 ± 5.0	73.3 ± 0.6
E	59.0 ± 2.1	35.8 ± 1.0
Cisplatin	24.8 ± 2.1	16.2 ± 2.4
Paclitaxel	>1.0	0.247 ± 0.04

**Figure 5:**
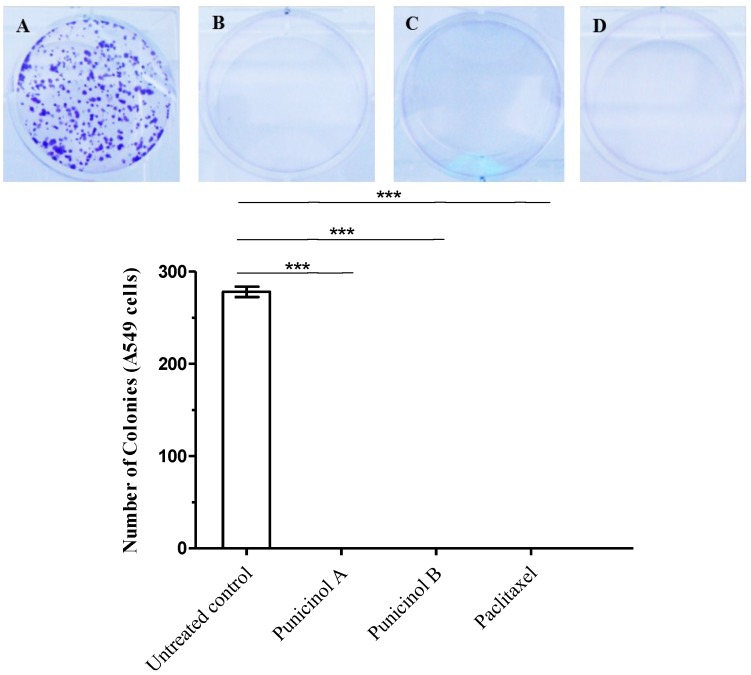
Clonogenic assay of A549 cells after exposure to punicinols A (**1**) and B (**2**) for 24 h. Clonogenic survival after 10 days was quantified by staining colonies using crystal violet. (**A**) Negative control. (**B**) Punicinol A (10 µM), (**C**) punicinol B (10 µM), and (**D**) paclitaxel (0.25 µM) which used as positive control. Data represent the mean ± standard deviations of three independent experiments. *** *p* < 0.0001 indicates statistically significant differences between the samples and the untreated control (ANOVA, Dunnett’s post test).

In order to evaluate the effectiveness of cytotoxic compounds over a longer treatment period, the clonogenic assay, an *in vitro* cell survival assay based on the capacity of a single cell to grow into a colony [31], was performed with punicinols A and B. It was shown to significantly inhibit cell growth, by 100%, when compared to the untreated control. This potent inhibitory effect was equal to that caused by paclitaxel (Figure 5). These data suggest that after 24 h of treatment, tumor cells do not return to proliferation after at least 10 days, showing the efficacy of these compounds over a longer treatment period, and also that that A549 cells are not resistant to them. The data from this study are novel and suggest that the isolated compounds may inhibit the growth of tumor cells for long periods of time.

The potential interaction of (punicinols A and B) with paclitaxel was evaluated by the methodology described by Chou (2006) [32]. The results were obtained in triplicate, and synergism was described as the combination index (CI). In this assay, both compounds showed synergism with paclitaxel, especially punicinol A, which presented a very strong synergism at the lowest tested concentrations (0.31 µM-punicinol A and 0.008 µM-paclitaxel). These results suggest that when combined with paclitaxel, punicinols A and B have high potential to induce cancer cell death. Punicinol A was more effective when combined with paclitaxel, due to the lower CI values (Table 4 and Table 5).

**Table 4:** Synergistic cytotoxic effects of the combinations of punicinol A (**1**) with paclitaxel in A549 cells.

Compounds Combination Ratio	Punicinol A (µM)	Paclitaxel (µM)	Experimental CI Values	Description (Graded Symbols)
4 × IC_50_	40	1.000	1.218	Moderate antagonism (++)
2 × IC_50_	20	0.500	1.248	Moderate antagonism (++)
1 × IC_50_	10	0.250	0.519	Synergism (+++)
0.5 × IC_50_	5	0.125	0.485	Synergism (+++)
0.25 × IC_50_	2.5	0.063	0.346	Synergism (+++)
0.125 × IC_50_	1.25	0.031	0.180	Strong synergism (++++)
0.062 × IC_50_	0.625	0.016	0.116	Strong synergism (++++)
0.031 × IC_50_	0.31	0.008	0.095	Very Strong synergism (+++++)

CI = combination index, a quantitative measure calculated by Calcusyn Software. This index quantiﬁes the interaction between the tested compounds as described in [32]. In detail, CI from 0.10–0.30 means strong synergism, 0.30–0.70 means synergism, 0.70–0.85 means moderate synergism, and 0.85–0.90 means slight synergism.

**Table 5:** Synergistic cytotoxic effects of the combinations of punicinol B (**2**) with paclitaxel in A549 cells.

Compounds Combination ratio	Punicinol B (µM)	Paclitaxel (µM)	Experimental CI values	Description (graded symbols)
4 × IC_50_	40	1.000	0.735	Moderate Synergism (++)
2 × IC_50_	20	0.500	0.633	Synergism (+++)
1× IC_50_	10	0.250	0.922	Additive effect (±)
0.5 × IC_50_	5	0.125	1.024	Additive effect (±)
0.25 × IC_50_	2.5	0.063	6.381	Strong antagonism (++++)
0.125 × IC_50_	1.25	0.031	3.191	Antagonism (−−−)
0.062 × IC_50_	0.625	0.016	1.595	Antagonism (−−−)
0.031 × IC_50_	0.31	0.008	0.799	Synergism (+++)

CI = combination index, a quantitative measure calculated by the CalcuSyn Software. This index quantiﬁes the interaction between the tested compounds [32]. In detail, CI values of 0.10–0.30 signify strong synergism, 0.30–0.70 signify synergism, 0.70–0.85 signify moderate synergism, and 0.85–0.90 signify slight synergism.

The polyoxygenated steroids previously isolated from octocorals of the *Leptogorgia* genus have shown promising cytotoxicity against different cancer cell lines. However, those compounds, are structurally different from the compounds described herein. In the previous study, the important features related to cytotoxicity were the α,β unsaturated ketone at ring A and the presence of a hydroxyl at C-16 instead of a carbonyl group in that position [16,21].

In order to discuss the structure–activity relationship of punicinols A-E, other steroids with similar skeleton were considered for comparison. Carvalho and co-workers (2011) discussed the structure-activity relationship (SAR) of polyoxygenated steroids, obtained by chemical and enzymatic syntheses, and showed that compounds with an acetyloxy group at position 6β displayed the best cytotoxic results when tested against colon cancer HT-29, LAMA-84, HepG2, A549, PC3, and MCF-7 cell lines [33]. Additionally, the presence of a free hydroxy group at position 3β seems to be crucial for the cytotoxic activity. The hydroxy group at position 7β and the acetyloxy group at position 4β also improve selectivity and cytotoxicity, and the OH in 4β position decreases it [33].

Other studies with compounds isolated from gorgonians describe the importance of the side chain structure in tumor cell growth inhibitory activity. A decrease in activity was observed in the case of oxidations at C-25, oxidation of the 6β hydroxyl to ketone, and in the presence of a double bond at C-7 [30,34].

Therefore, the cytotoxic effects of punicinols A–E are consistent with these studies, since these compounds present the main characteristics described above. In our study, the importance of the side chain was also observed, since the compounds with double bonds on the side chain (punicinols C, D and E) were less cytotoxic than punicinols A and B.

## 3. Experimental Section

### 3.1. General

Infrared spectra (IR) were obtained in an IR Prestige-21 FTIR-8400 S (Shimadzu) using KBr. NMR spectra were obtained on an Bruker Avance 2 spectrometer (^1^H: 500 MHz, 1^3^C: 125 MHz) in CDCl_3_. Chemical shifts are given in δ (ppm) using TMS as internal standard. The 2D experiments (HSQC, HMBC, COSY, NOESY) were performed using standard Bruker pulse sequences. High-resolution ESI (MS and MS2) mass spectra were obtained on a Bruker MicrOTOF-Q II spectrometer, in positive ion mode. During the isolation procedures, chromatographic separations were performed on silica gel 60 columns (0.040–0.063 mm), TLC analysis was performed on silica gel F254 plates (Merck^®^ Darmstadt, Germany and Silicycle^®^, Quebec, Canada). HPLC separations were performed on a Thermo Separation^®^ liquid chromatographer equipped with an UV-Vis detector (S100), and a refractive index detector (Thermo separation products Refractor Monitor^®^ IR) using a preparative ODS-A column (YMC-Pack ODS-A, SH343-5, 5 µm, 20 × 250 mm) with a flow rate of 5 mL·min^−1^.

### 3.2. Biological Material Collection

The octocoral *Leptogorgia punicea* was collected by SCUBA at a depth of 10–14 m off the coast of Aranhas Island (27°29'12"S; 48°21'37"W), Florianópolis, Santa Catarina State, South Brazil. The biological material was kept frozen at −20 °C. A voucher specimen was identified by Bárbara Segal and was deposited at the Cnidarian Collection of the Department of Ecology and Zoology (Universidade Federal de Santa Catarina, Florianópolis, SC, Brazil).

### 3.3. Extraction and Isolation

The frozen octocoral (2 kg) was extracted exhaustively with ethanol. The ethanol extract was concentrated under reduced pressure and the resulting dried extract was resuspended in distillated water and partitioned three times with *n*-hexane (HF fraction). The HF fraction (1.6 g) was subjected to chromatographic column on silica gel (hexane-ethyl acetate-methanol gradient). Fractions 109 and 110 (130 mg) were then submitted to another silica gel column eluted with chloroform: ethyl acetate (1:1). The fractions containing the main components were pooled (F1, 22.6 mg) and assayed for cytotoxic activity. Fraction F1 was purified by reversed-phase HPLC using methanol: Milli-Q water (90:10) as eluent, and a flow rate of 5 mL/min to yield the major compounds **1** (4.8 mg) and **2** (3.5 mg) at Rt of 20 min and 26 min, respectively, as well as the minor compounds **3** (1.0 mg), **4** (0.8 mg) and **5** (0.6 mg).

**Compound 1: 6β-acetoxycholestane-3β,5α,11β-triol**: White powder, mp 105–108 °C.

IR (KBr) υ cm^−1^: 3442, 1714, 1371, 1259, 1163, 1037.

HR ESI MS: *m/z* 496.3964 [M + NH_4_]^+^ (100), (calc. for C_29_H_54_NO_5_^+^ 496.3997); CID-experiment (CE 15 eV, Argon) on [M + NH_4_]^+^ *m/z* 496.00: *m/z* 478.3891 [M + NH_4_ − H_2_O]^+^ (calc. for C_29_H_52_NO_4_ 478.3891); *m/z* 461.3619 [M + H − H_2_O]^+^ (calc. for C_29_H_49_O_4_ 461.3625) and *m/z* 443.3518 [M + H − 2H_2_O]^+^ (calc. for C_29_H_47_O_3_ 443.3520) and *m/z* 383.3311 [M + H − 3H_2_O − C_2_H_2_O]^+^ (calc. for C_27_H_43_O 383.3308).

NMR: Table 1 and Table 2 and Supplementary Information.

**Compound 2: 6β-acetoxycholestane-3β,5α,18-triol**: White powder, mp 110–111 °C.

IR (KBr) υ cm^−1^: 3442, 1714, 1377, 1257, 1244, 1165, 1033.

HR ESI MS: *m/z* 501.3540 ([M + Na]^+^ (100), (calc. for C_29_H_50_NaO_5_^+^ 501.3550); CID-MS/MS experiment (CE 26 eV, Argon) on [M + Na]^+^ *m/z* 501.00: *m/z* 459.3505 [M + Na-C_2_H_2_O]^+^ (calc. for C_29_H_47_O_4_ 459.3469) and *m/z* 441.3331 [M + Na − C_2_H_2_O − H_2_O]^+^ (calc. for C_29_H_45_O_3_ 441.3363).

NMR: Table 1 and Table 2 and Supplementary Information.

**Compound 3: 6β-acetoxycholest-*22E*-ene-3β,5α,11β-triol**: White powder, mp 102–104 °C.

HR ESI MS: *m/z* 499.3406 [M + Na]^+^ (100), (calc. for C_29_H_48_NaO_5_^+^ 499.3394), *m/z* 494.3854 [M + NH_4_]^+^ (calc. for C_29_H_52_NO_5_^+^ 494.3840); CID-MS/MS experiment (CE 10 eV, Argon) on [M + NH_4_]^+^ *m/z* 494.00: *m/z* 476.3735 [M + NH_4_ − H_2_O]^+^ (calc. for C_29_H_50_NO_4_ 476.3734), *m/z* 459.3463 [M + H − H_2_O]^+^ (calc. for C_29_H_47_O_4_ 459.3469) and *m/z* 381.3142 [M + H − C_2_H_2_O − 3H_2_O]^+^ (calc. for C_27_H_41_O 381.3152).

NMR: Table 1 and Table 2 and Supplementary Information.

**Compound 4: 24-nor-6β-acetoxycholest-22*E*-ene-3β,5α,11β-triol**: White powder, mp 109–111 °C.

HR ESI MS: *m/z* 485.3261 [M + Na]^+^ (100) (calc. for C_28_H_46_NaO_5_^+^ 485.3237), *m/z* 480.3690 [M + NH_4_]^+^ (calc. for C_28_H_50_NO_5_^+^ 480.3684); CID-MS/MS experiment (CE 10 eV, Argon) on [M + NH_4_]^+^ *m/z* 480.00: *m/z* 462.3546 [M + NH_4_ − H_2_O]^+^ (calc. for C_28_H_48_NO_4_ 462.3578), *m/z* 445.3271 [M + H − H_2_O]^+^ (calc. for C_28_H_45_O_4_ 445.3312) and *m/z* 367.2970 [M + H − C_2_H_2_O − 3H_2_O]^+^ (calc. for C_26_H_38_O 367.2995).

NMR: Table 1 and Table 2 and Supplementary Information.

**Compound 5: 6β-acetoxycholest-*22E*-ene-3β,5α,18-triol**: White powder, mp 107–108 °C.

HR ESI MS: *m/z* 499.3394 [M + Na]^+^ (100) (calc. for C_29_H_48_NaO_5_^+^ 499.3394), *m/z* 494.3844 [M + NH_4_]^+^ (calc. for C_29_H_52_NO_5_^+^ 494.3840); CID-MS/MS experiment (CE 10 eV, Argon) on [M + NH_4_]^+^ *m/z* 494.00: *m/z* 476.3715 [M + NH_4_ − H_2_O]^+^ (calc. for C_29_H_50_NO_4_ 476.3734), *m/z* 459.3428 [M + H − H_2_O]^+^ (calc. for C_29_H_47_O_4_ 459.3469) and *m/z* 381.3119 [M + H − C_2_H_2_O − 3H_2_O]^+^ (calc. for C_27_H_41_O 381.3152).

NMR: Table 1 and Table 2 and Supplementary Information.

### 3.4. Biological Activity

#### 3.4.1. Cell Culture

Non-small cell lung cancer cells (A549 cells) were obtained from ATCC^®^ (CCL-185). These cells were cultured in Minimum Essential Medium (MEM, Cultilab, Campinas, Brazil) supplemented with 10% fetal bovine serum and maintained at 37 °C in a humidified 5% CO_2_ atmosphere.

#### 3.4.2. Sulforhodamine B Assay

The cytotoxic effects of the samples were measured by the sulforhodamine B assay [35]. Briefly, A549 cells were seeded at a density of 1 × 10^4^ cells/100 µL/well in 96-well plates (TPP, Switzerland) and incubated for 24 h. The medium was then removed and the samples were added at eight different concentrations (ratio 1:2), and incubated for 24 and 48 h. The cells were then fixed with 10% trichloroacetic acid for 60 min and washed with distilled water. After fixation, the cells were stained with sulforhodamine B solution (Sigma-Aldrich, St. Louis, MO, USA) for 30 min, and the excess dye was removed by washing repeatedly with 1% acetic acid solution. The protein-bound dye was dissolved in 10 mM Tris base solution for optical density (OD) determination at 510 nm using a microplate reader (Spectra Max, Molecular Devices, Sunnyvale, CA, USA). All experiments were performed in triplicate. The cytotoxic concentration of each sample for 50% of cells (CC_50_) was calculated from the linear regression analysis of the concentration-response curves plotted for each tested sample, according to the following equation:

$$\text{Growth inhibition}\;=\;(\text{O}{\text{D}}_{control}\;-\;\text{O}{\text{D}}_{treated})/(\text{O}{\text{D}}_{control}\;-\;\text{O}{\text{D}}_{blank})\;\times \;100\%$$

#### 3.4.3. Synergistic Effects of Punicinols A and B in Combination with Paclitaxel

The potential synergistic effects of punicinols A and B in combination with paclitaxel were evaluated by the sulforhodamine B assay, according to the experimental design proposed by Chou (2006) [32]. Briefly, A549 cells were seeded at a density of 1 × 10^4^ cells/100 µL/well in 96-well plates (TPP, Switzerland) and incubated for 24 h. After 24 h, each sample alone or in combination was tested at a fixed ratio of its corresponding IC_50_ values (*i.e.*, at IC_50_ × 0.25, ×0.5, ×1, ×2, and ×4) for 48 h. The degree of interaction between punicinols A and B and paclitaxel was calculated through the combination index (CI) equation, based on the median-effect principle of the mass-action law, using the Calcusyn software (version 2.1, Biosoft^®^). According to the CI theorem, CI values <1, =1, and >1 indicate synergism, additive effect and antagonism, respectively.

#### 3.4.4. Cell Cycle Analysis by Flow Cytometry

A549 cells were seeded at a density of 5 × 10^5^ cells/well in six-well plates (TPP, Switzerland) and incubated for 24 h. Next, the cells were treated with samples for 24 h at a concentration of 10 µM. After treatment, cells were harvested by trypsinization and centrifuged at 500× g for 5 min. The resulting pellets were washed with PBS (phosphate buffered saline) and the cells were fixed in ice-cold 70% ethanol and stored at 4 °C for 30 min. After fixation, the cells were washed with 2% Bovine Serum Albumin solution, centrifuged for 10 min, and ressuspended with PBS containing RNase A, propidium iodide and glycerol at 37 °C for 30 min. Flow cytometry analyses were carried out on a FACS Canto II instrument (Becton Dickinson, Heildelberg, Germany), followed by determination of the cell population in each phase of the cell cycle, using the Flowing 2.5.0 Software (University of Turku, Finland).

#### 3.4.5. Clonogenic Assay

The clonogenic assay is an *in vitro* survival assay of cells based on the ability of a single cell to grow into a colony. This assay was performed according to Franken and co-workers (2006) [31], with minor modifications. Initially, 2.5 × 10^2^ A549 cells/well were seeded in six-well plates (TPP, Switzerland) and incubated for 24 h. Thereafter, A549 cells were treated with punicinols A and B at a concentration of 10 µM for 24 h. The medium was removed and each well was washed with PBS and received fresh medium supplemented with 10% fetal bovine serum and incubated for 10 days. After this period, the serum-supplemented medium was removed and each well was washed again with PBS, and cell monolayers were stained with 0.5% crystal violet (w/v). The stained colonies formed were counted using a stereomicroscope (Olympus, Tokyo, Japan). The number of colonies formed represents the average of three independent experiments ± standard deviations.

## 4. Conclusions

Chemical investigation of the octocoral *Leptogorgia punicea* collected from South Brazil led to the isolation and structural elucidation of five new polyoxygenated steroids (punicinols A–E).

The major isolated compounds: 6β-acetoxycholestane-3β,5α,11β-triol (punicinol A) and 6β-acetoxycholestane-3β,5α,18-triol (punicinol B) showed relevant cytotoxic activity against A549 cells, while punicinols C, D and E, which present a double bond on the side chain, were less cytotoxic. Cell cytotoxic effects induced by these compounds may be explained by changes in the cell cycle, through the arrest of the Sub G0/G1 (punicinol A) and G2M (punicinol B) phases. These results show, as in other previous studies, that the nature of the side chain may play an important role in the cytotoxic activity. Furthermore, the importance of the β-acetyloxy group attached to position 6, and the free hydroxyl group at position 3, are also supported. Punicinols A and B also showed important effects on clonogenic potential of A549 cells, completely inhibiting the growth of tumor cells after 24 h of treatment and 10 days for monitoring cell proliferation. Additionally, punicinols A and B combined with paclitaxel, demonstrated a better induction of cancer cell *in vitro* compared with the individual compounds, and this study indicates synergistic effects of punicinols A–B and paclitaxel in tumor chemotherapy.

## Supplementary Files

Supplementary File 1
