# Supplementary material for: Polyoxygenated Steroids from the Octocoral Leptogorgia punicea and in Vitro Evaluation of Their Cytotoxic Activity
Source: Mar Drugs. 2014 Dec 4;12(12):5864–80. doi: 10.3390/md12125864 (PMC4278206; doi:10.3390/md12125864)
Supplement: Supplementary File 1 [file marinedrugs-12-05864-s001.pdf]

Supplementary Information

Figure S1. ^{1}H NMR (500 MHz, $CDCl_3$) spectrum of punicinol A (**1**)
Figure S2. ^{13}C NMR (125 MHz, $CDCl_3$) spectrum of punicinol A (**1**)
Figure S3. HSQC-DEPT spectrum of punicinol A (**1**)
Figure S4. ^{1}H-^{1}H COSY spectrum of punicinol A (**1**)
Figure S5. HMBC spectrum of punicinol A (**1**)
Figure S6. NOESY spectrum of punicinol A (**1**)
Figure S7. ^{1}H NMR (500 MHz, $CDCl_3$) spectrum of punicinol B (**2**)
Figure S8. ^{13}C NMR (125 MHz, $CDCl_3$) spectrum of punicinol B (**2**)
Figure S9. HSQC-DEPT spectrum of punicinol B (**2**)
Figure S10. ^{1}H-^{1}H COSY spectrum of punicinol B (**2**)
Figure S11. HMBC spectrum of punicinol B (**2**)
Figure S12. NOESY spectrum of punicinol B (**2**)
Figure S13. ^{1}H NMR (500 MHz, $CDCl_3$) spectrum of punicinol C (**3**)
Figure S14. ^{13}C NMR (125 MHz, $CDCl_3$) spectrum of punicinol C (**3**)
Figure S15. HSQC-DEPT spectrum of punicinol C (**3**)
Figure S16. ^{1}H-^{1}H COSY spectrum of punicinol C (**3**)
Figure S17. HMBC spectrum of punicinol C (**3**)
Figure S18. NOESY spectrum of punicinol C (**3**)
Figure S19. ^{1}H NMR (500 MHz, $CDCl_3$) spectrum of punicinol D (**4**)
Figure S20. ^{13}C NMR (125 MHz, $CDCl_3$) spectrum of punicinol D (**4**)
Figure S21. HSQC-DEPT spectrum of punicinol D (**4**)
Figure S22. ^{1}H-^{1}H COSY spectrum of punicinol D (**4**)
Figure S23. HMBC spectrum of punicinol D (**4**)
Figure S24. NOESY spectrum of punicinol D (**4**)
Figure S25. ^{1}H NMR (500 MHz, $CDCl_3$) spectrum of punicinol E (**5**)
Figure S26. ^{13}C NMR (125 MHz, $CDCl_3$) spectrum of punicinol E (**5**)
Figure S27. HSQC-DEPT spectrum of punicinol E (**5**)
Figure S28. ^{1}H-^{1}H COSY spectrum of punicinol E (**5**)
Figure S29. HMBC spectrum of punicinol E (**5**)
Figure S30. NOESY spectrum of punicinol E (**5**)

Figure S1. ^{1}H NMR (500 MHz, $CDCl_3$) spectrum of punicinol A (**1**).

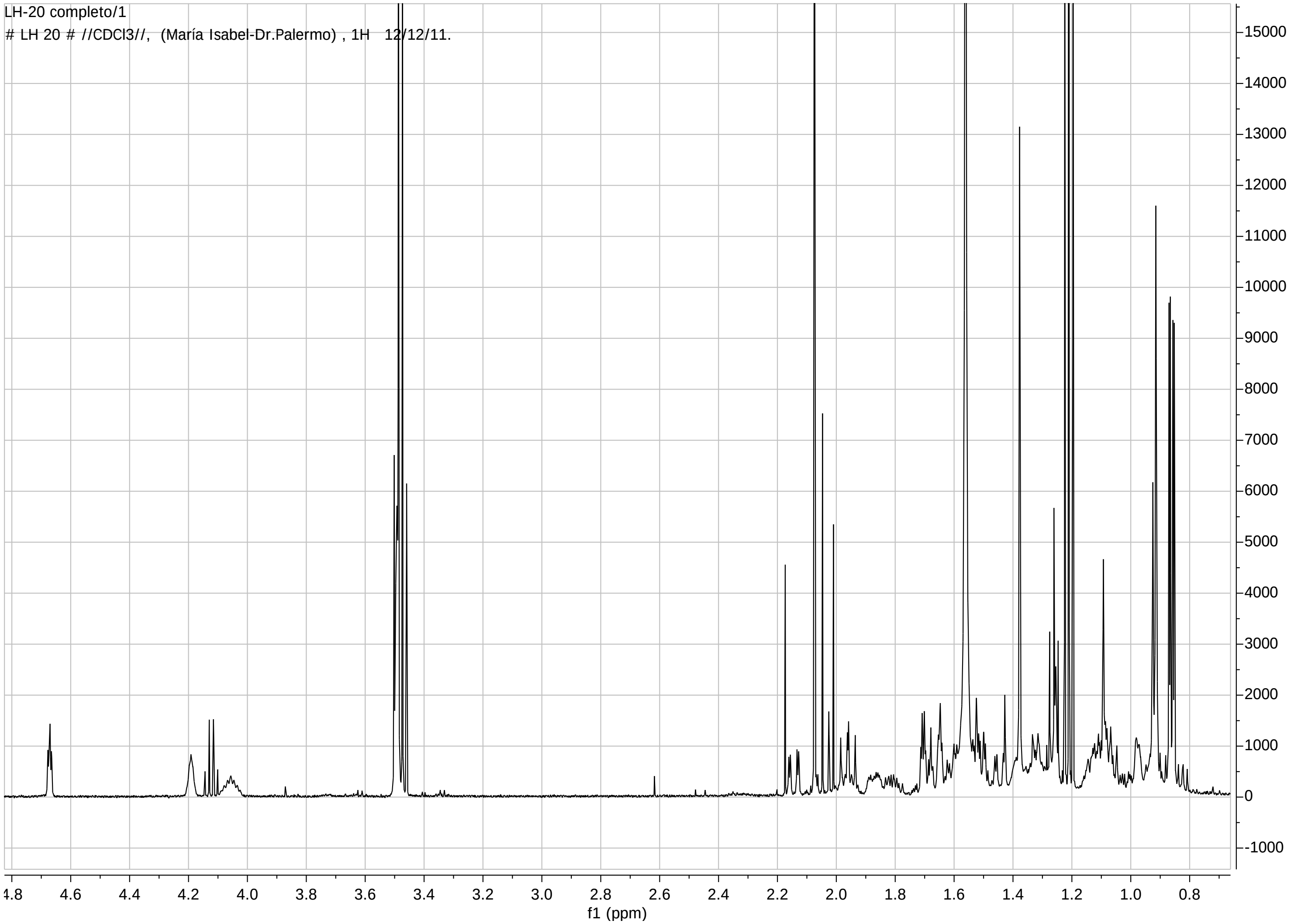

Figure S2. ^{13}C NMR (125 MHz, $CDCl_3$) spectrum of punicinol A (**1**).

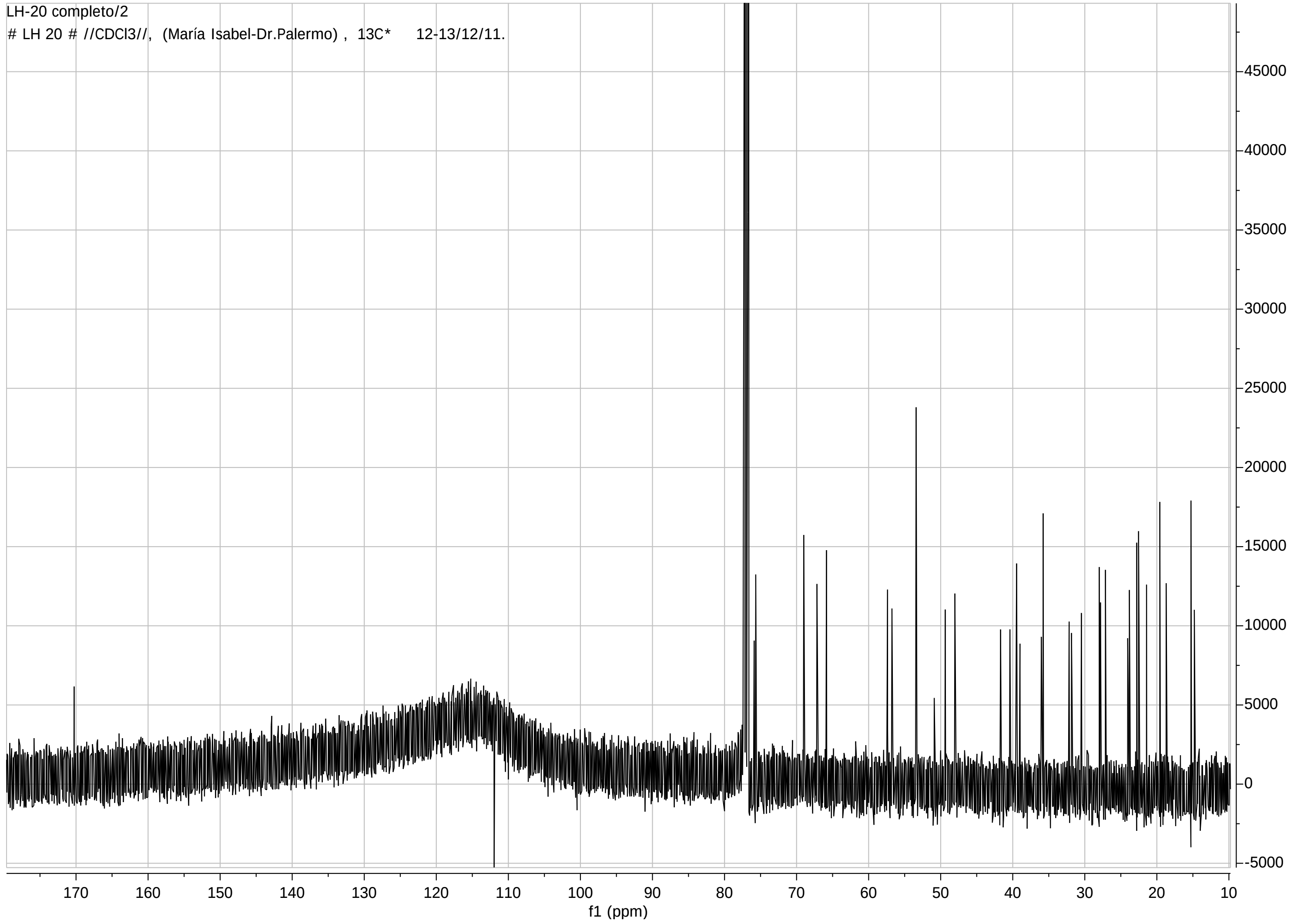

Figure S3. HSQC-DEPT spectrum of punicinol A (**1**).

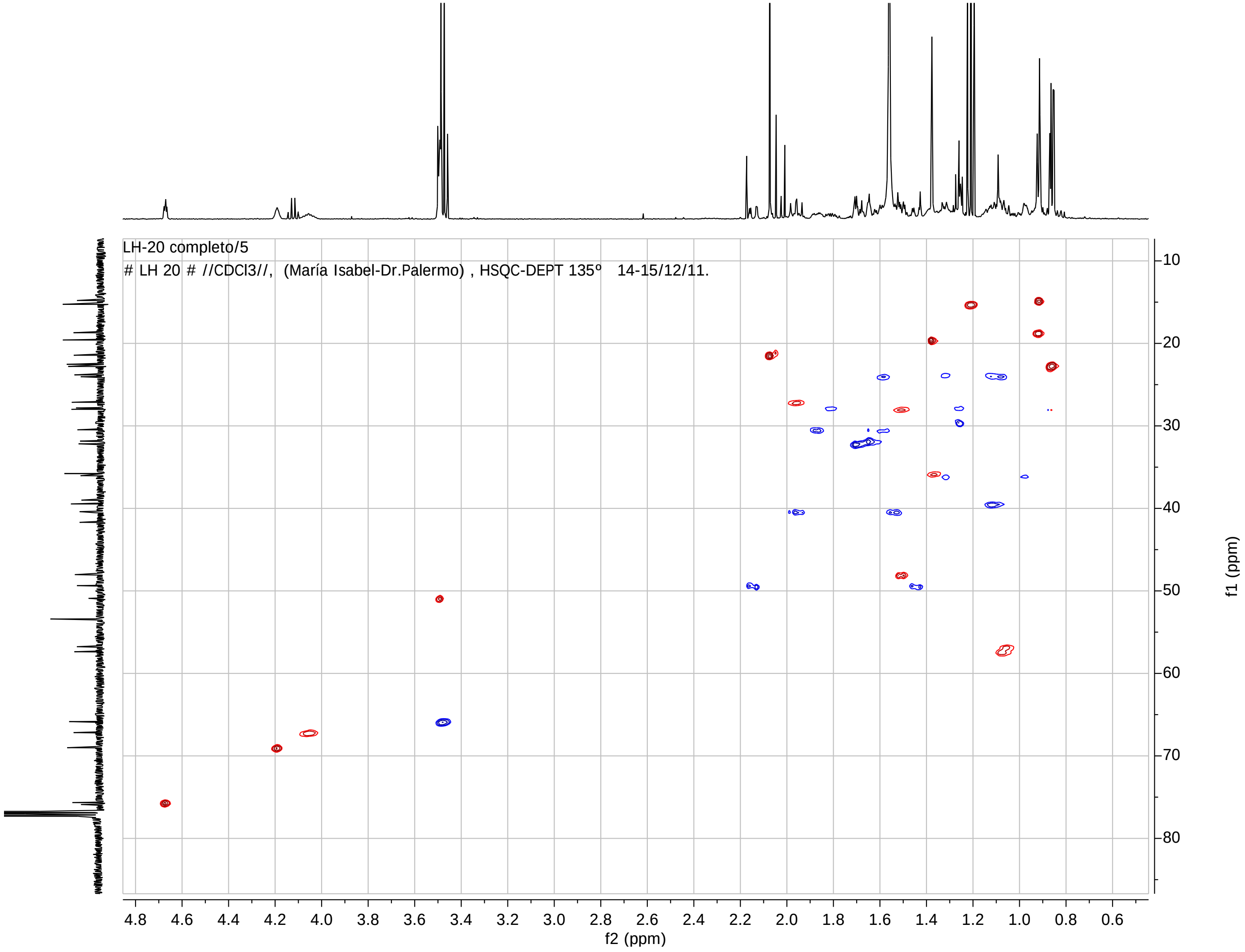

Figure S4. ^{1}H-^{1}H COSY spectrum of punicinol A (**1**).

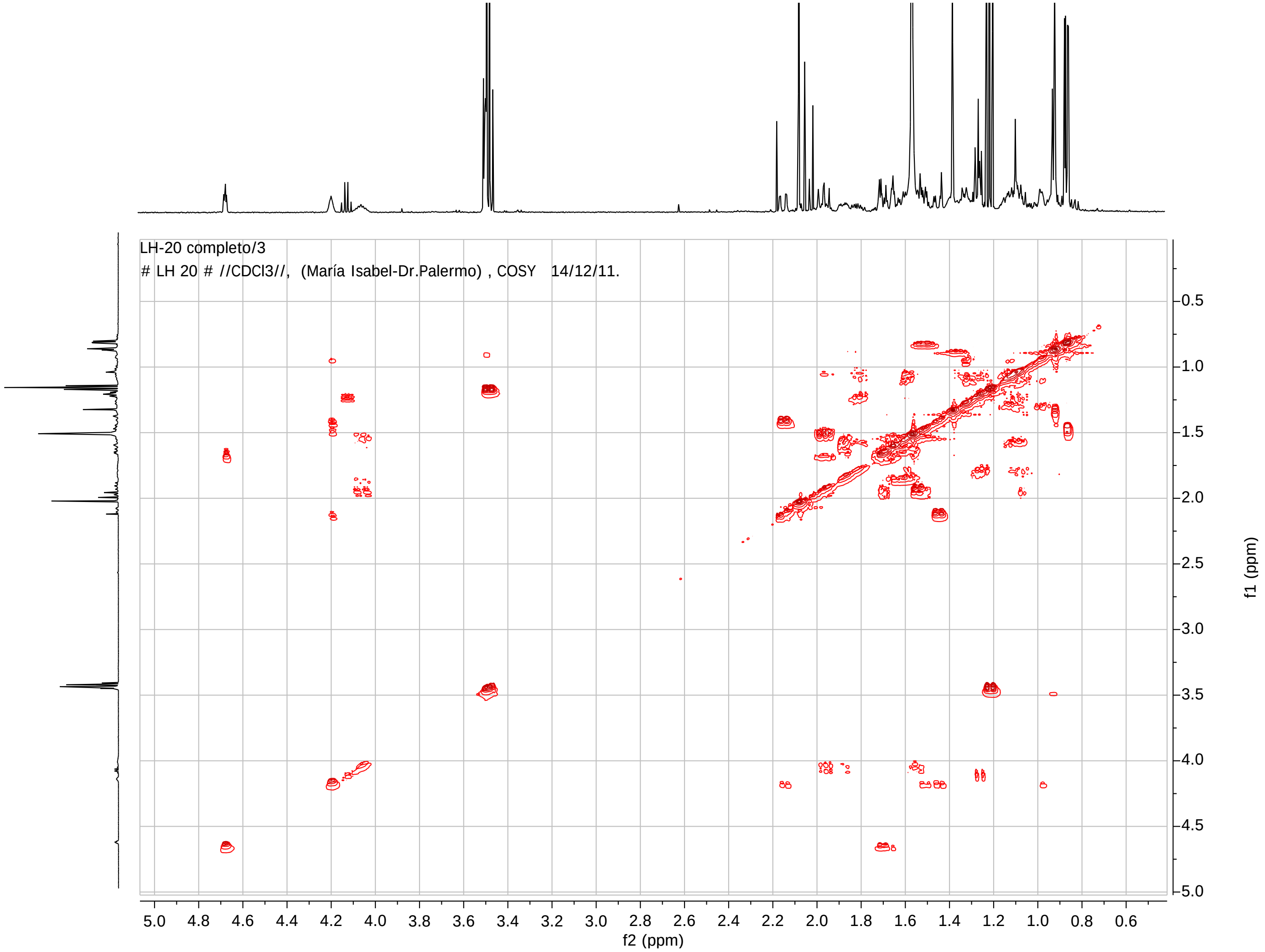

Figure S5. HMBC spectrum of punicinol A (**1**).

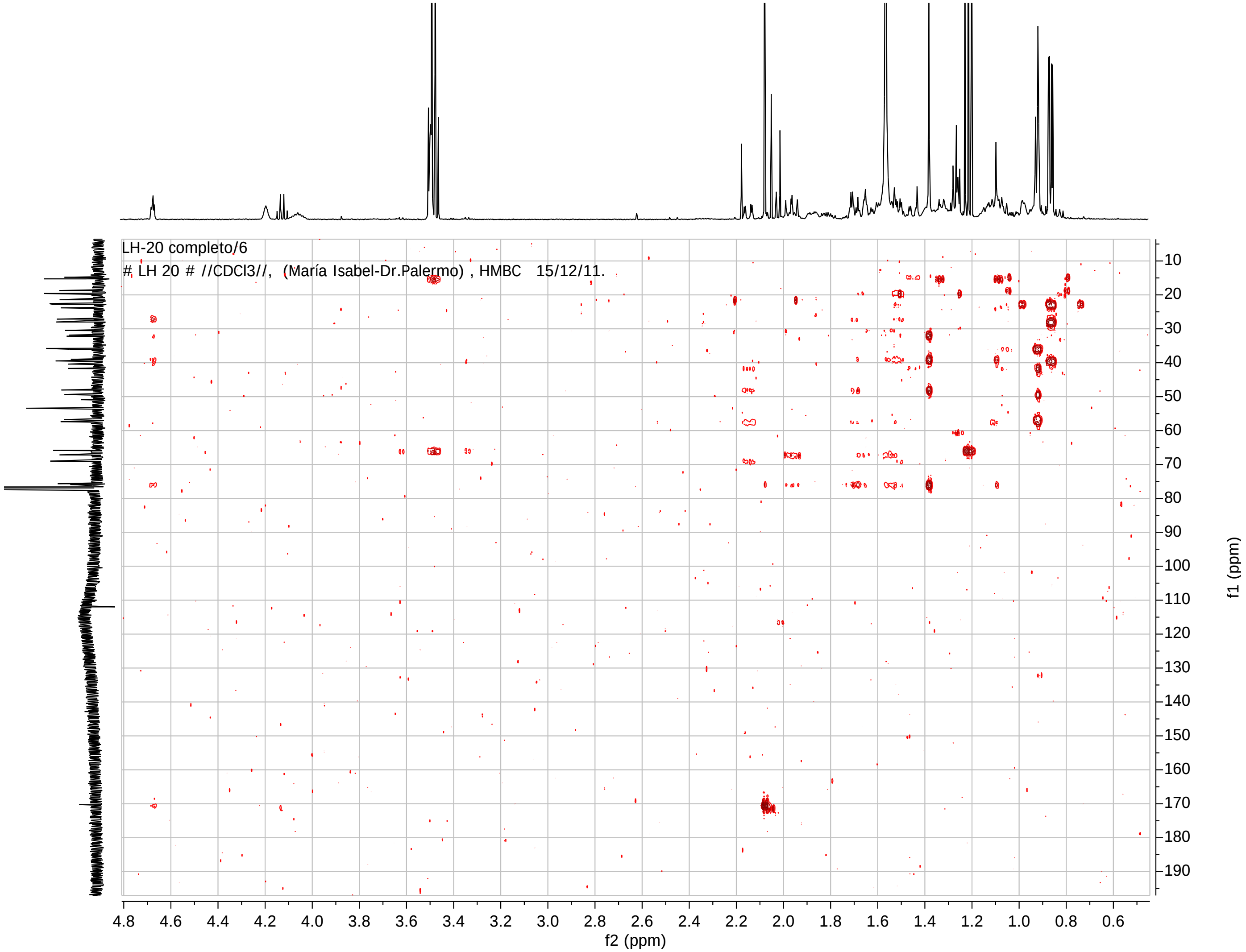

Figure S6. NOESY spectrum of punicinol A (**1**).

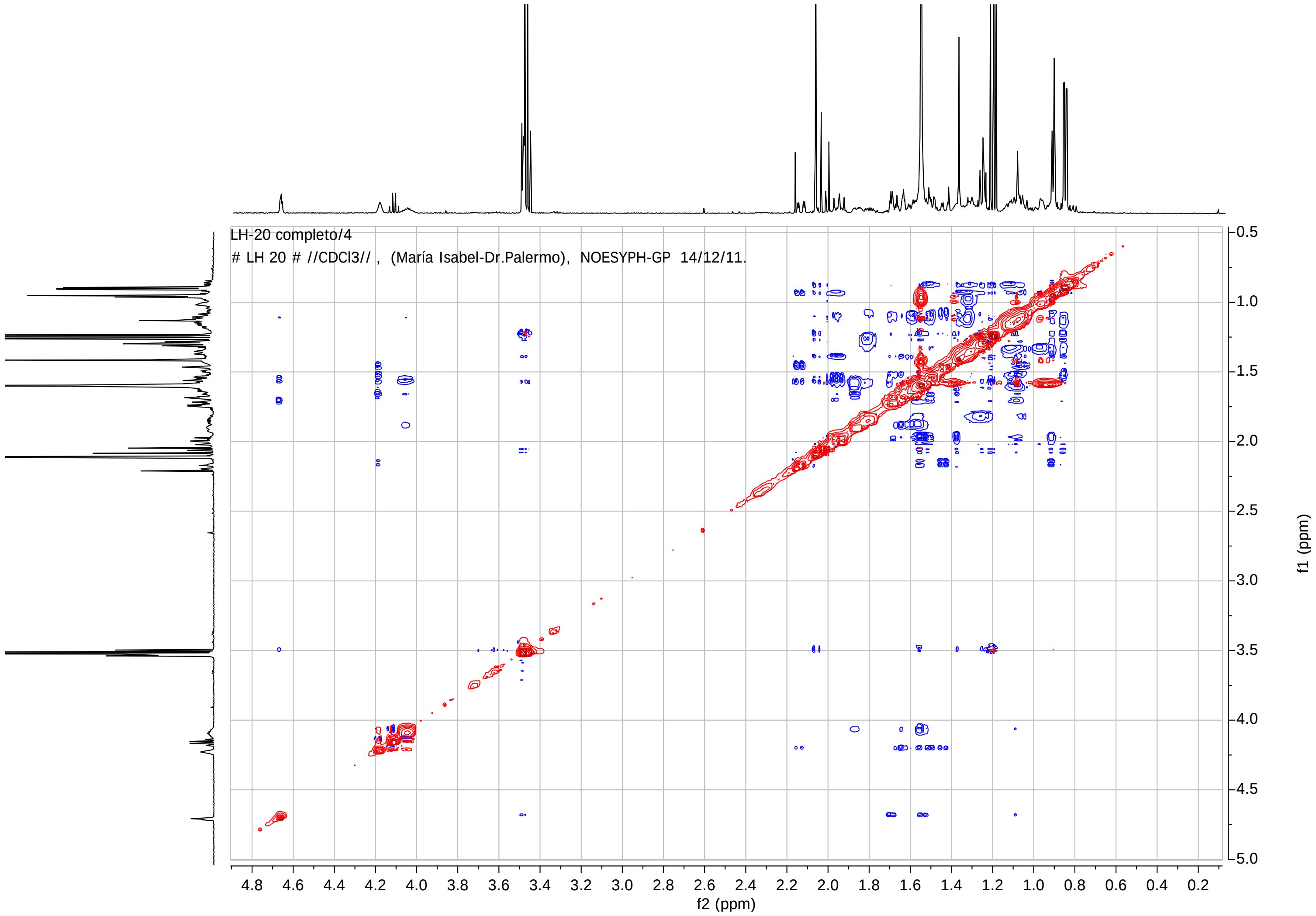

Figure S7. ^{1}H NMR (500 MHz, $CDCl_3$) spectrum of punicinol B (**2**).

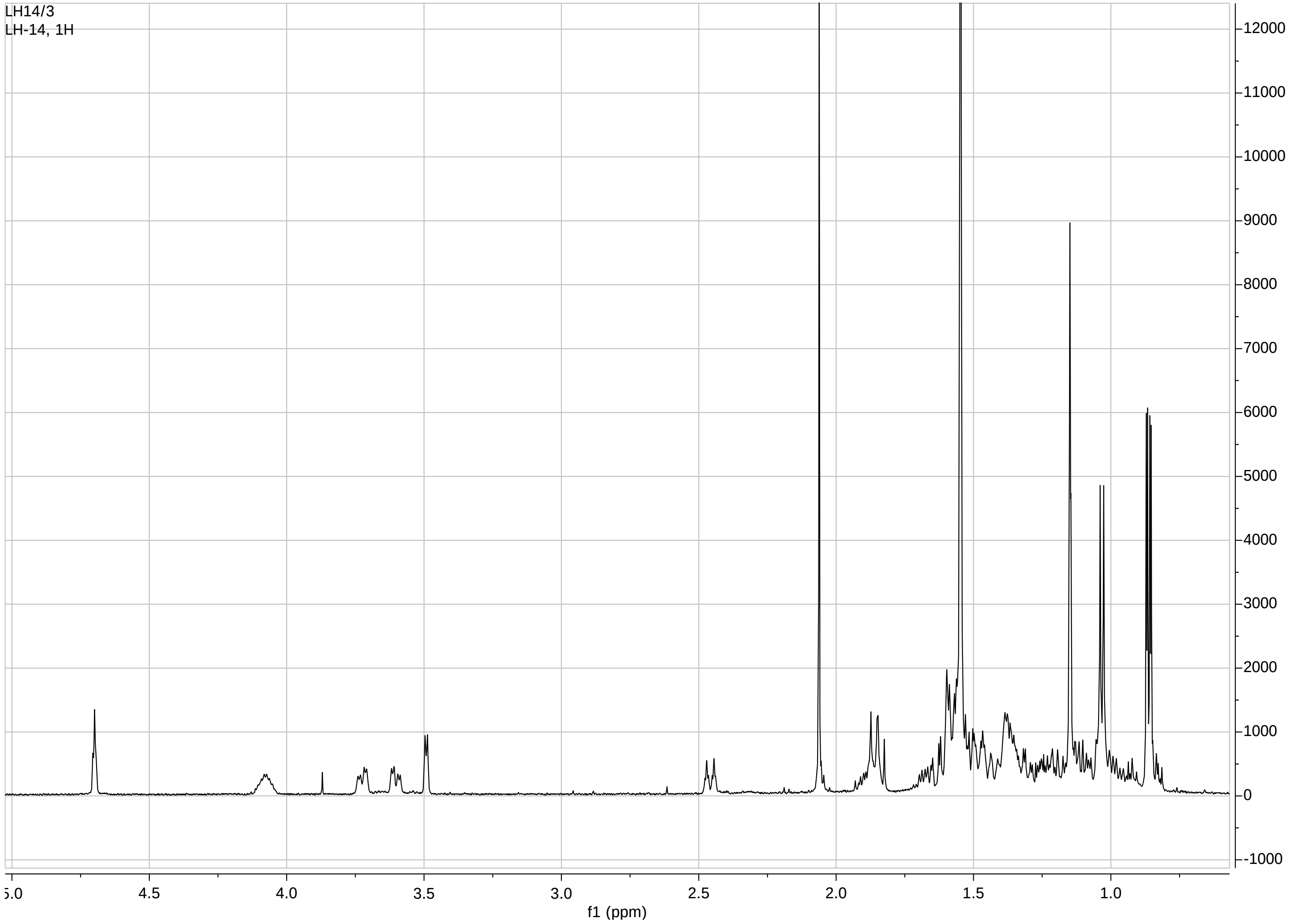

Figure S8. ^{13}C NMR (125 MHz, $CDCl_3$) spectrum of punicinol B (**2**).

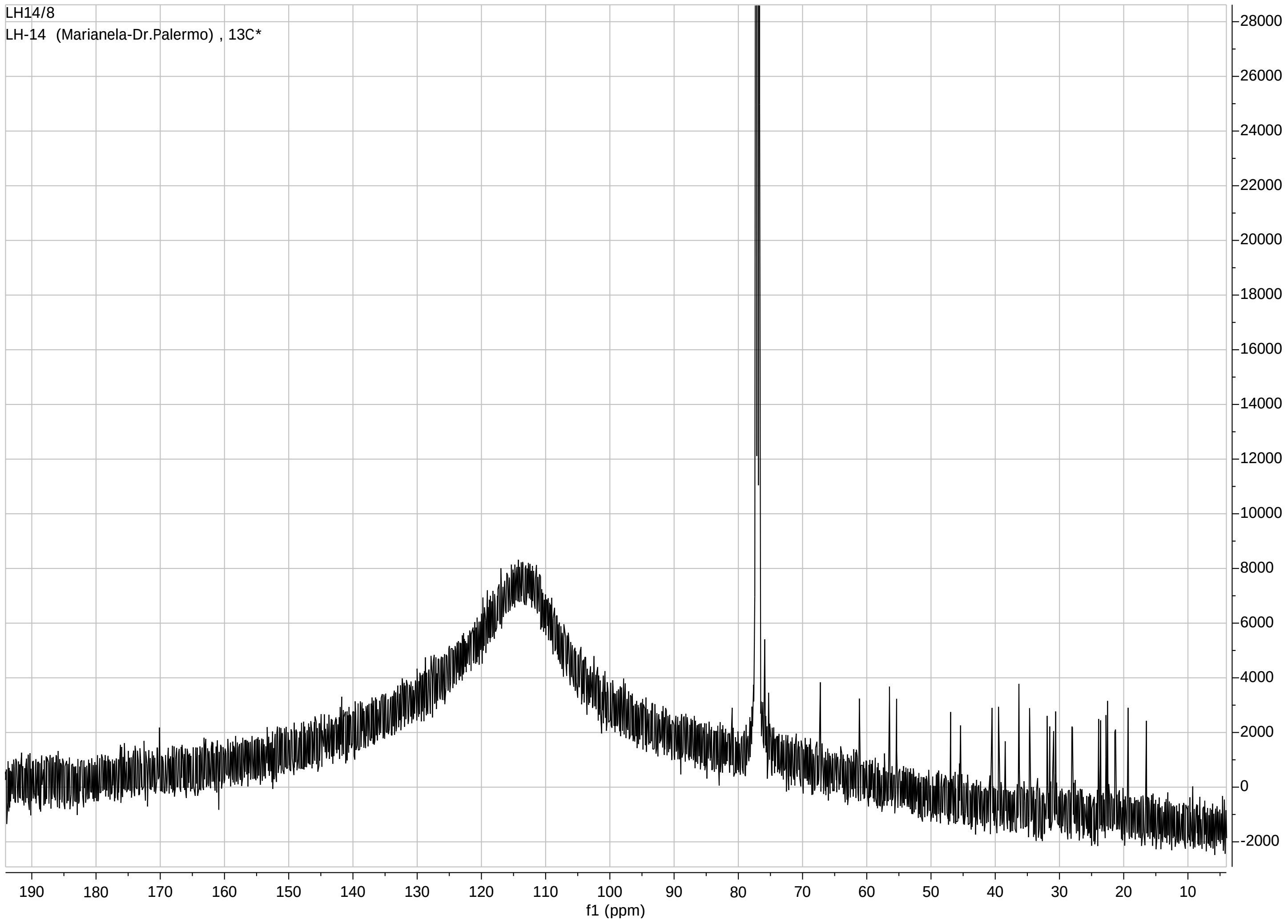

Figure S9. HSQC-DEPT spectrum of punicinol B (**2**).

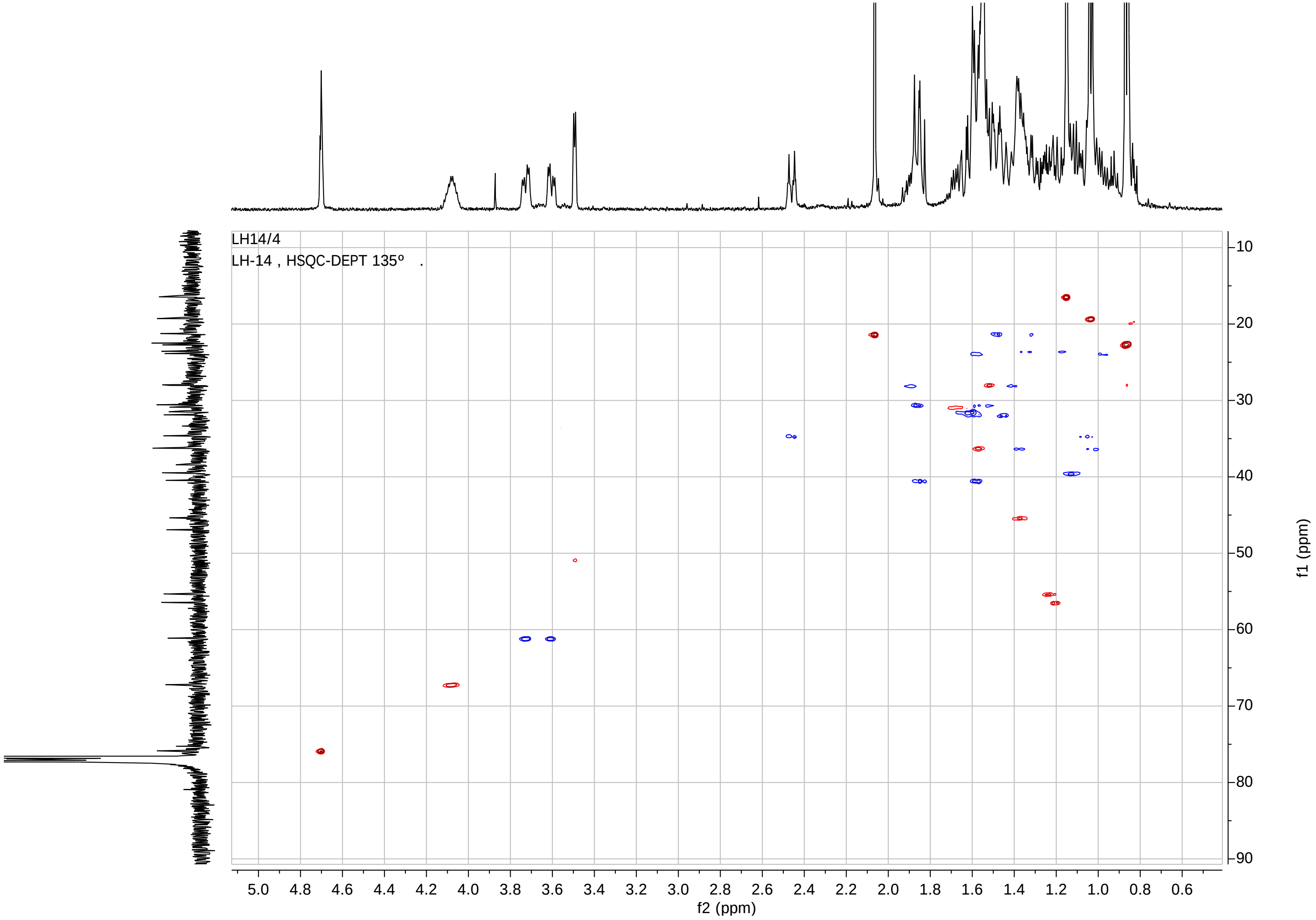

Figure S10. ^{1}H-^{1}H COSY spectrum of punicinol B (**2**).

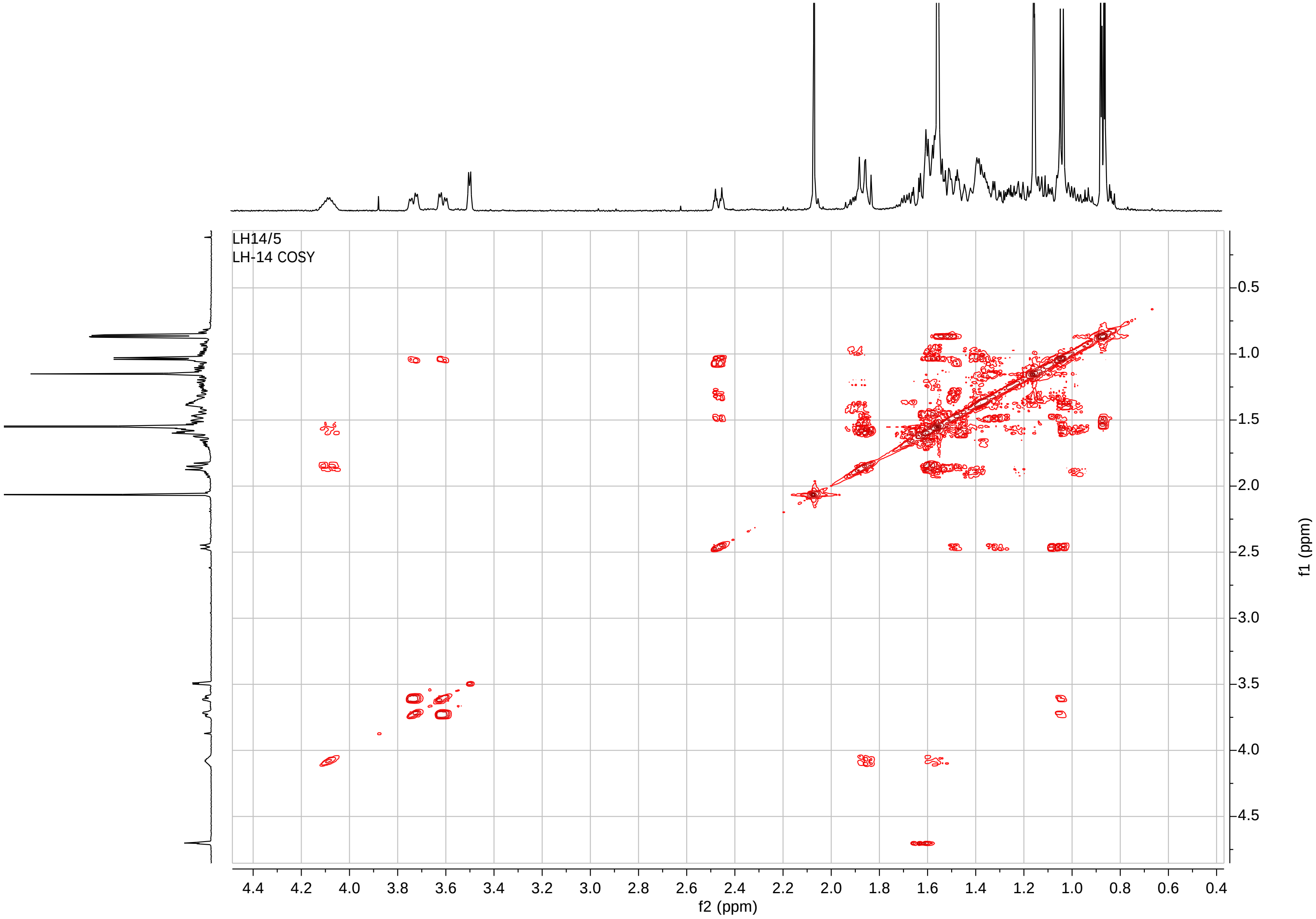

Figure S11. HMBC spectrum of punicinol B (**2**).

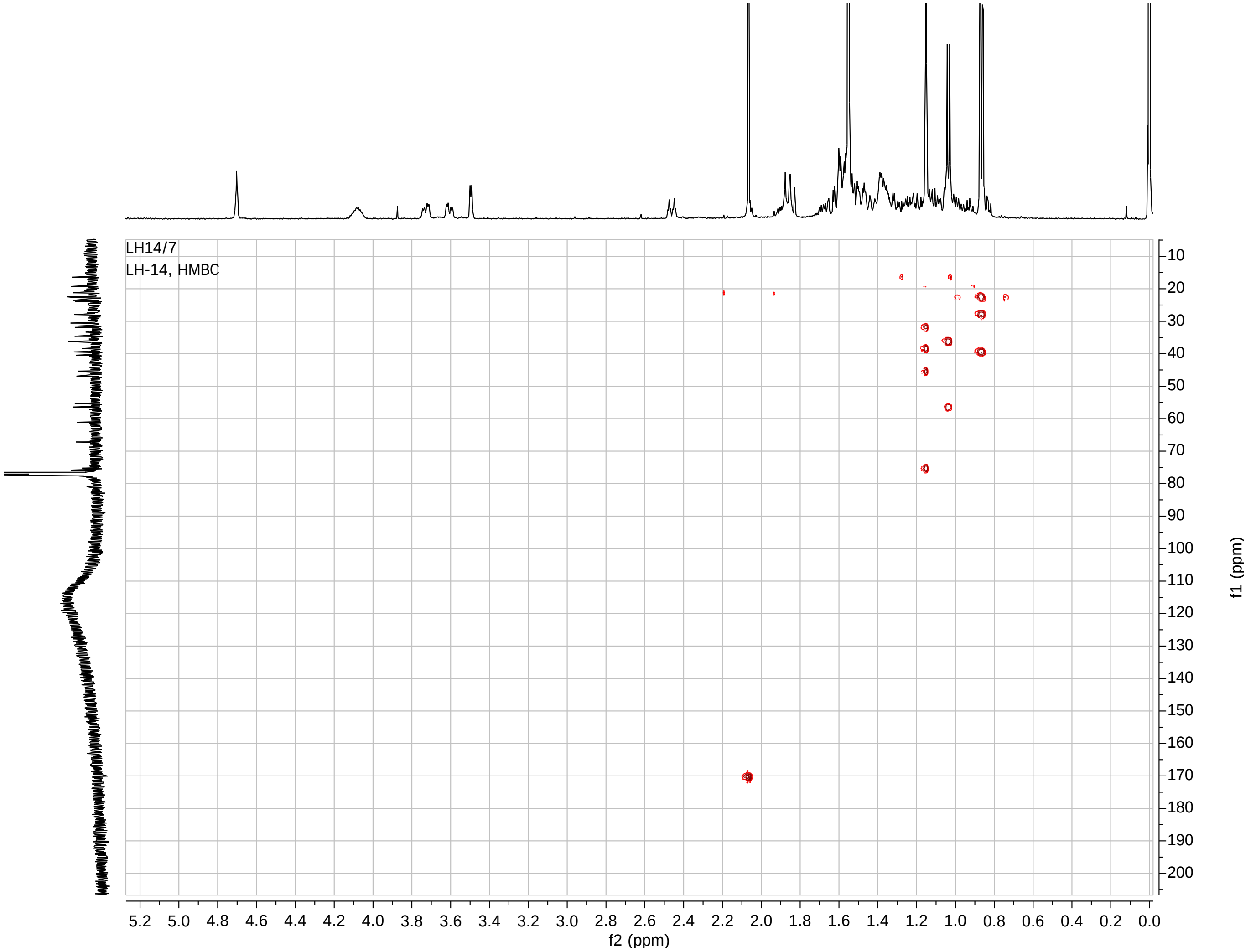

Figure S12. NOESY spectrum of punicinol B (**2**).

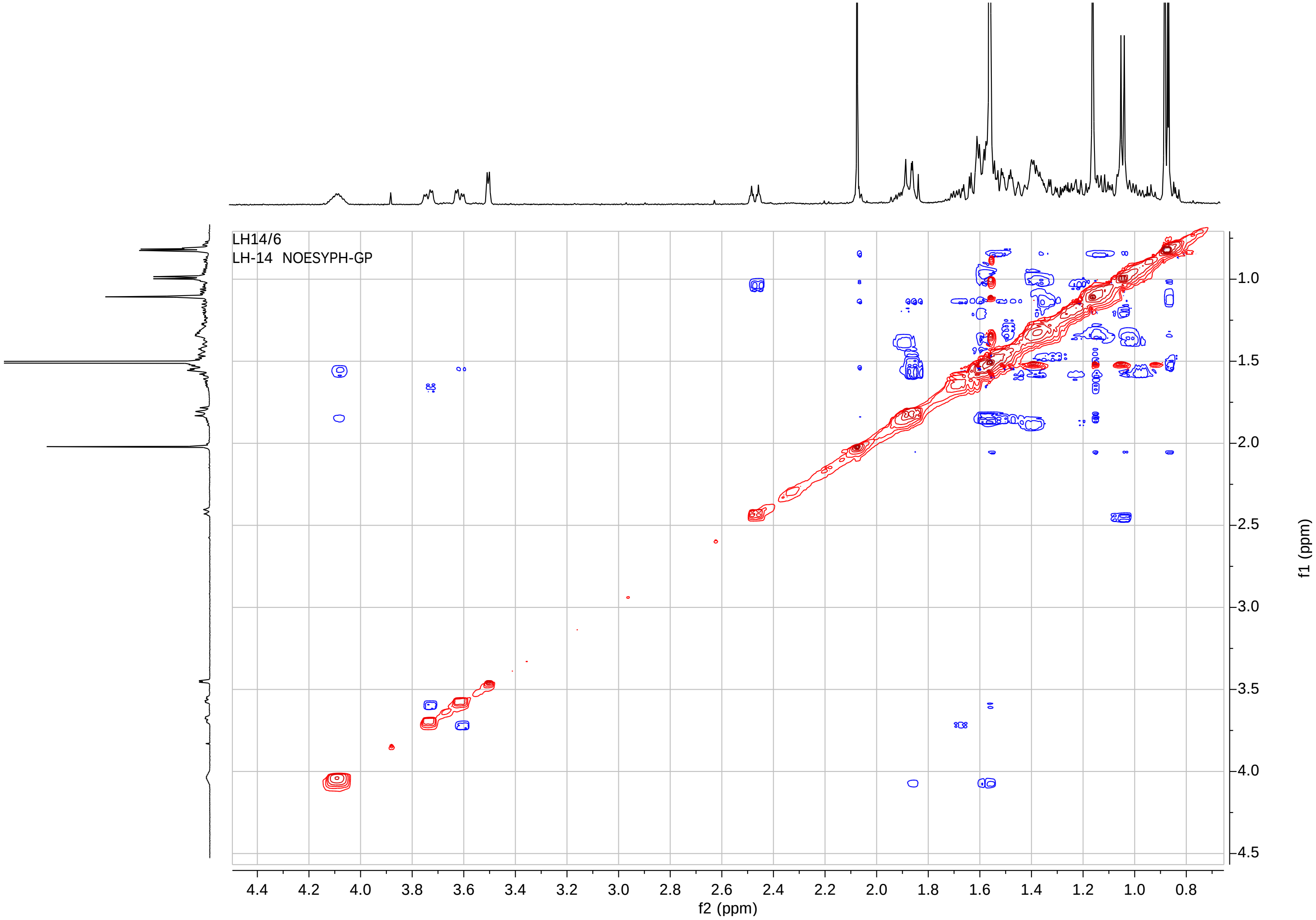

Figure S13. ^{1}H NMR (500 MHz, $CDCl_3$) spectrum of punicinol C (**3**).

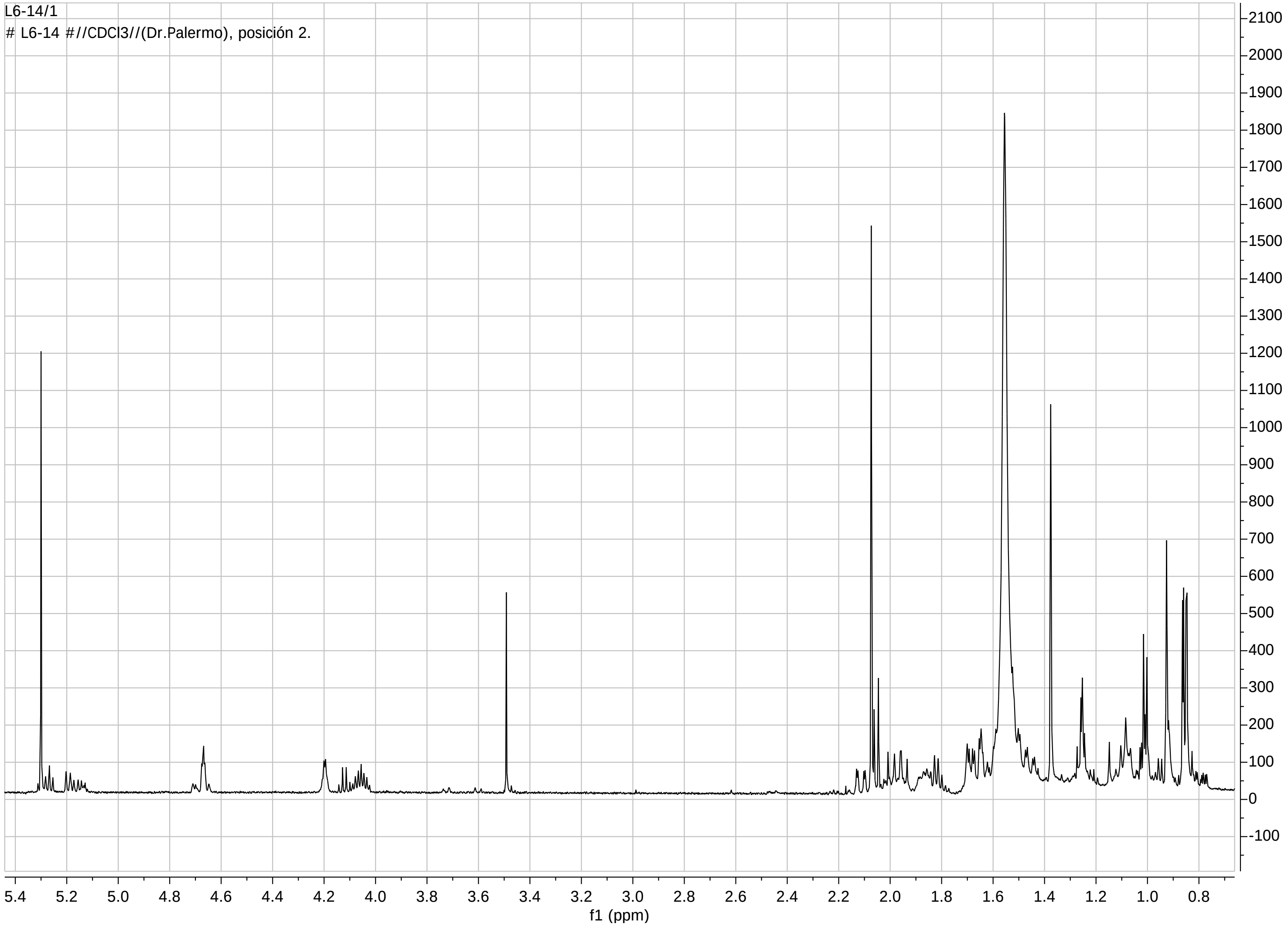

Figure S14. ^{13}C NMR (125 MHz, $CDCl_3$) spectrum of punicinol C (**3**).

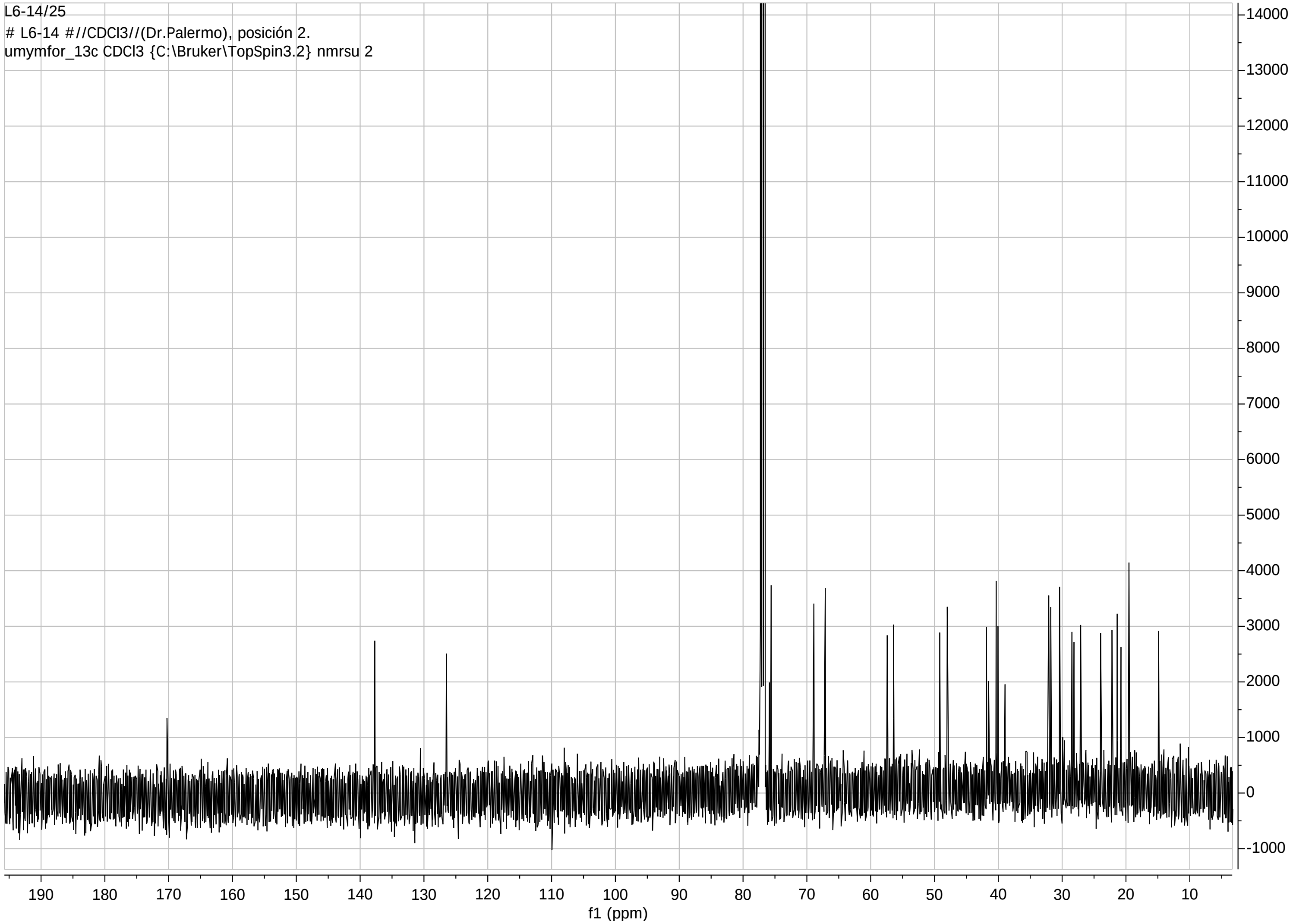

Figure S15. HSQC-DEPT spectrum of punicinol C (**3**).

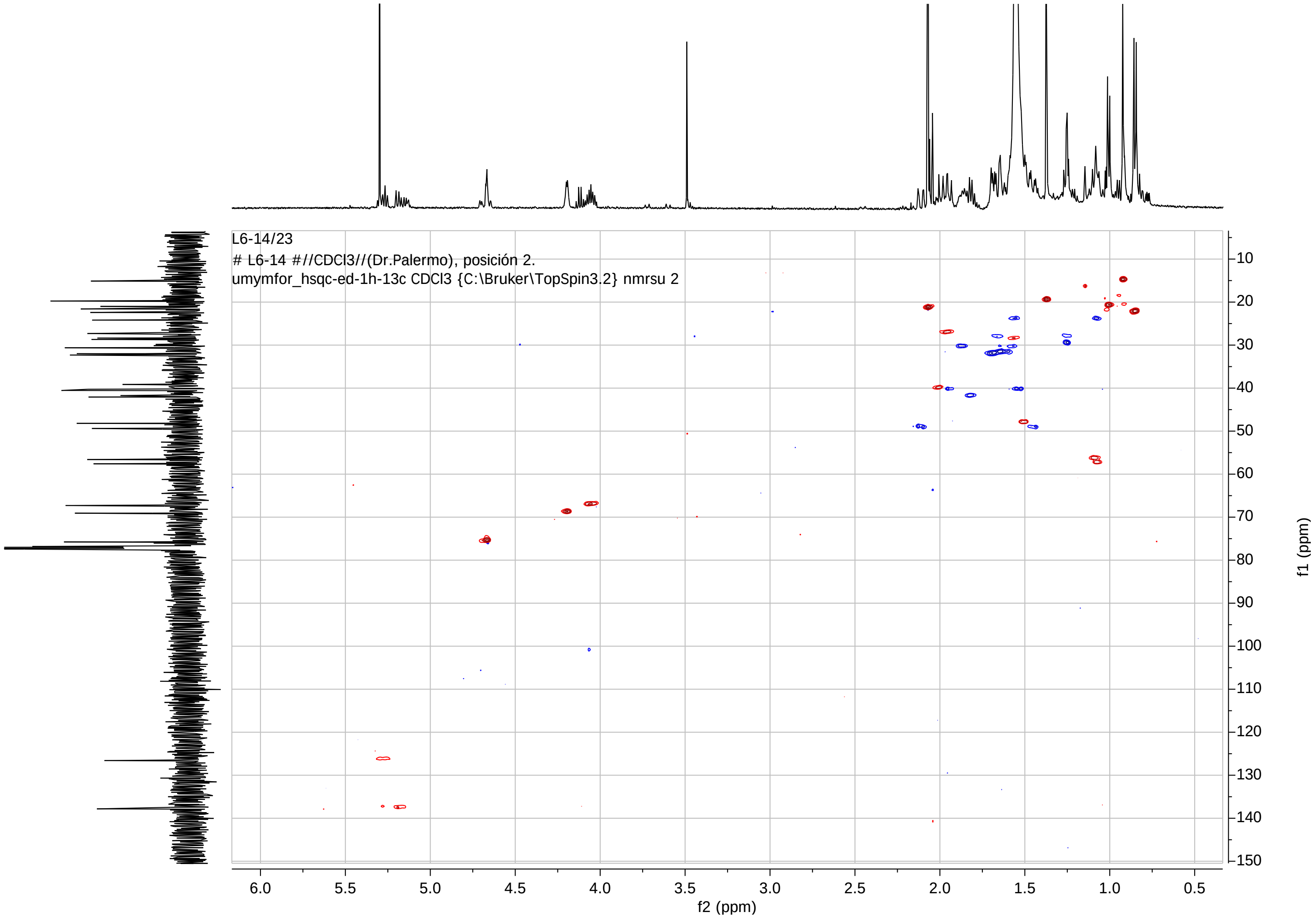

Figure S16. ^{1}H-^{1}H COSY spectrum of punicinol C (**3**).

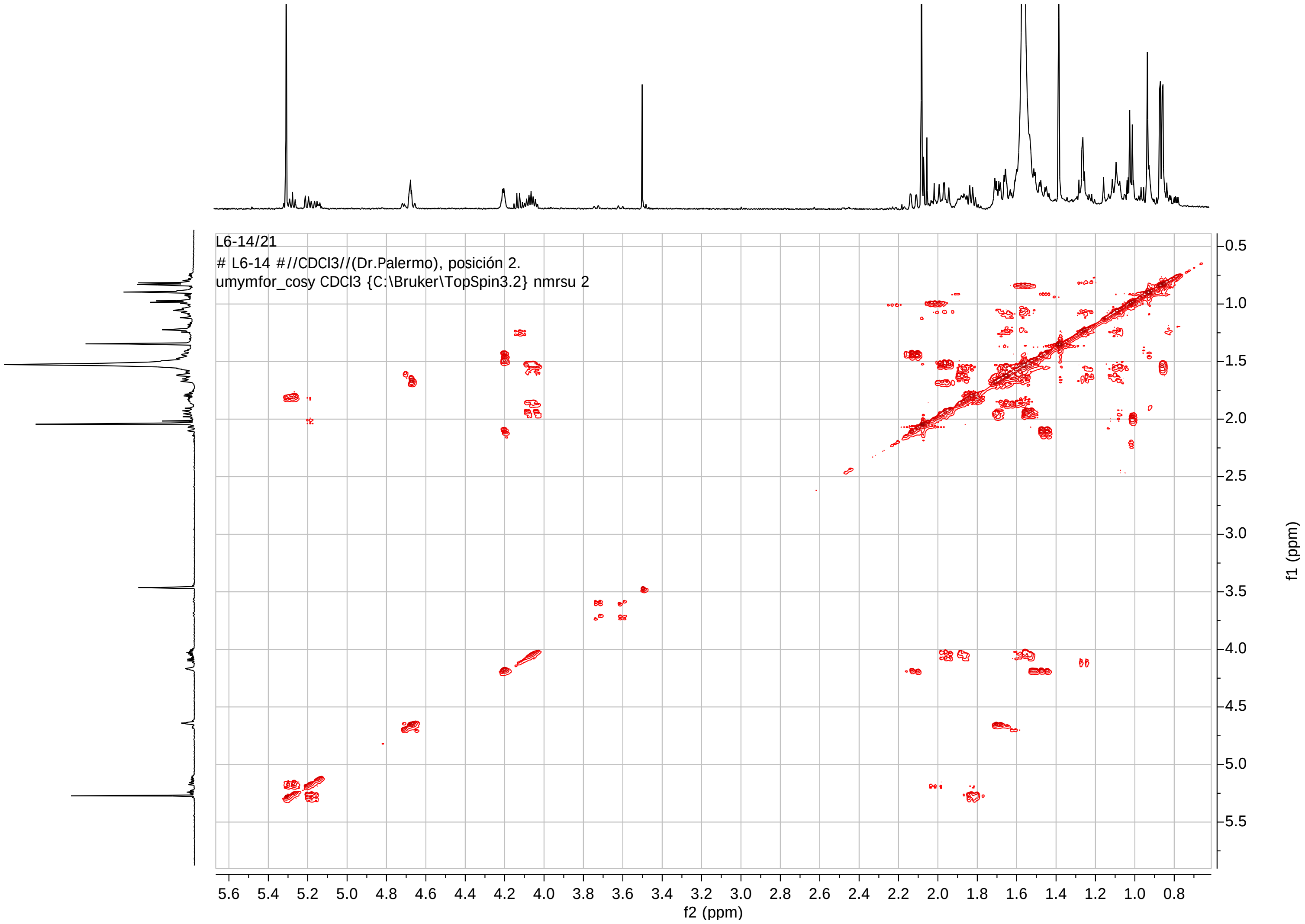

Figure S17. HMBC spectrum of punicinol C (**3**).

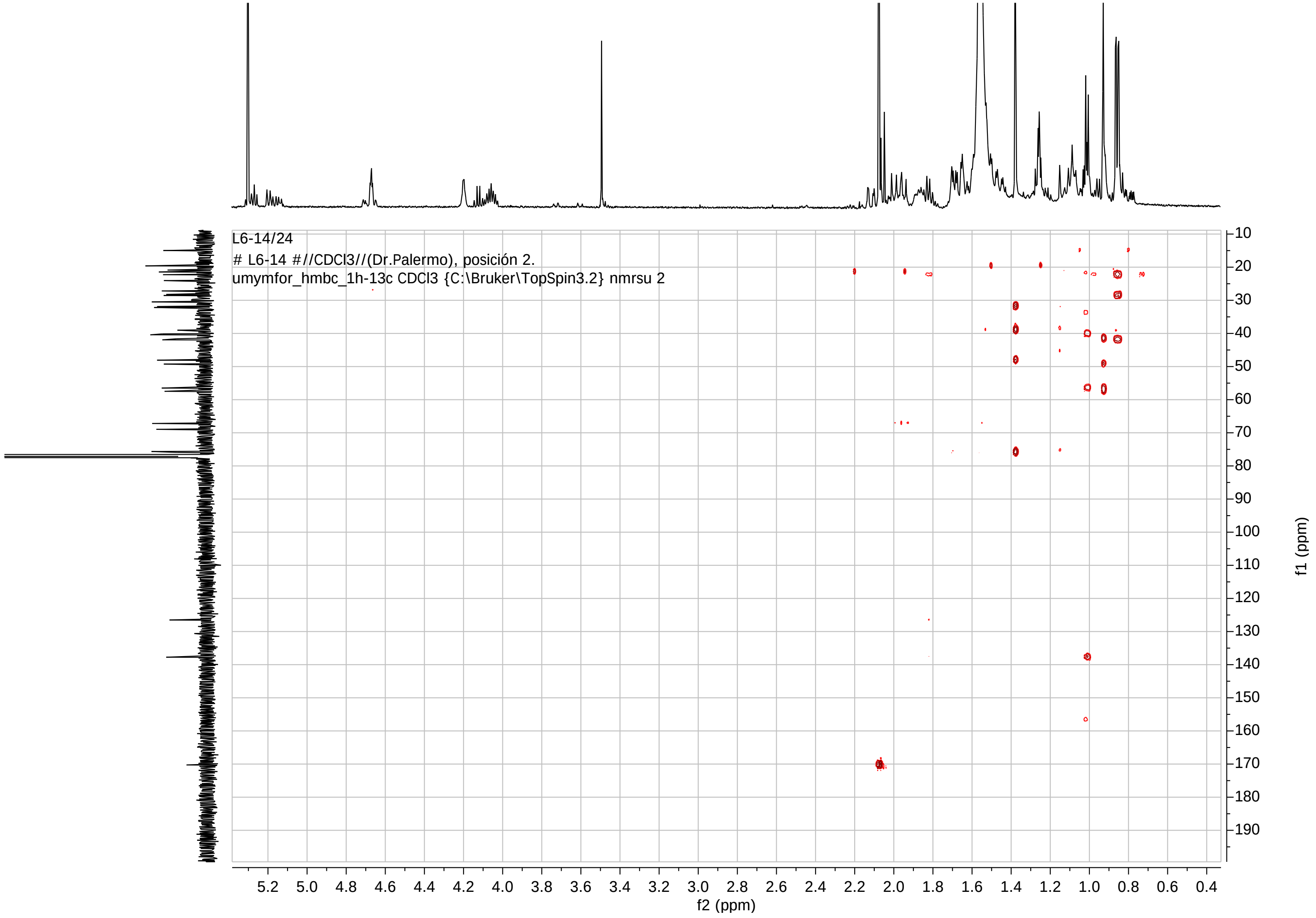

Figure S18. NOESY spectrum of punicinol C (**3**).

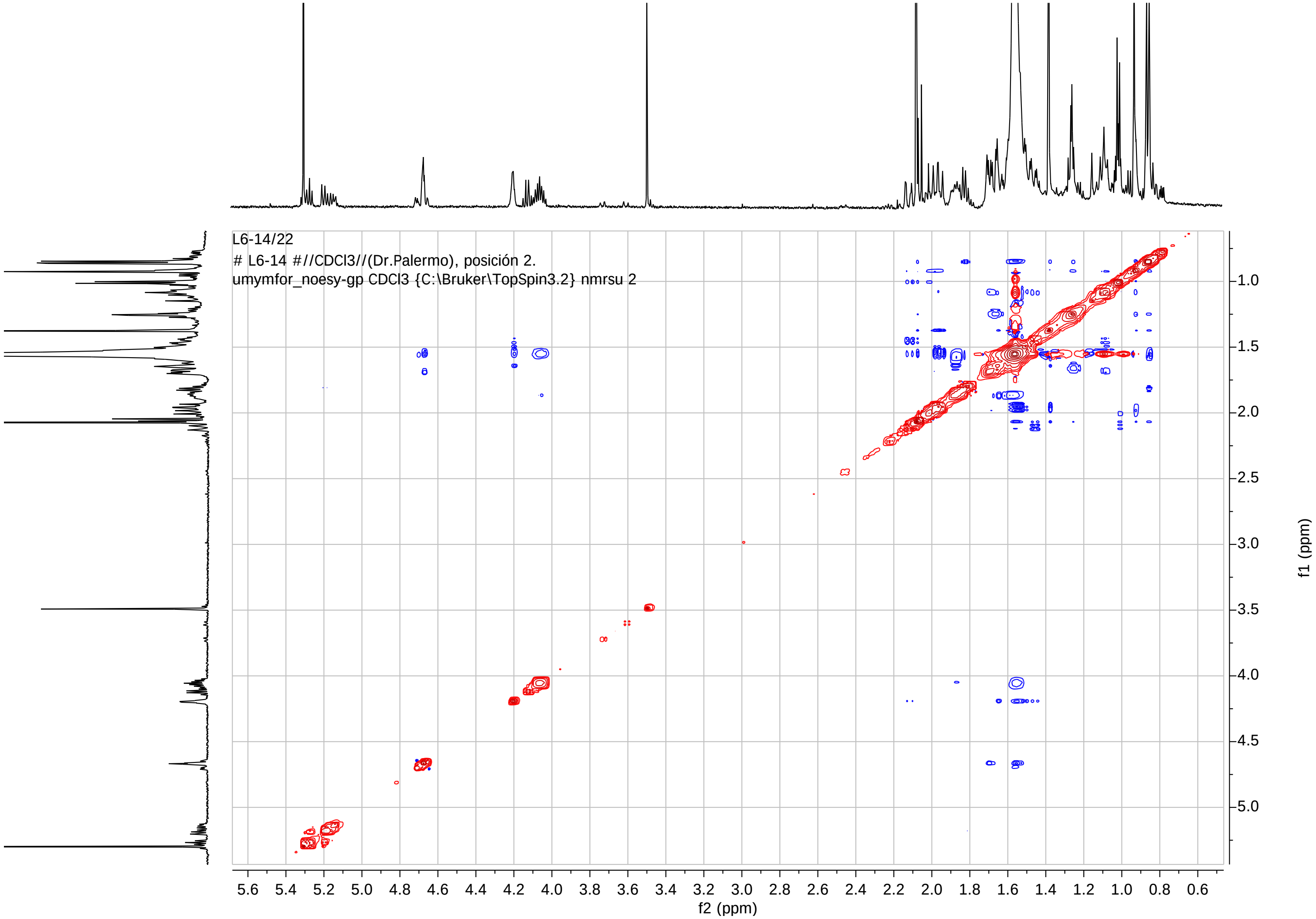

Figure S19. ^{1}H NMR (500 MHz, $CDCl_3$) spectrum of punicinol D (**4**).

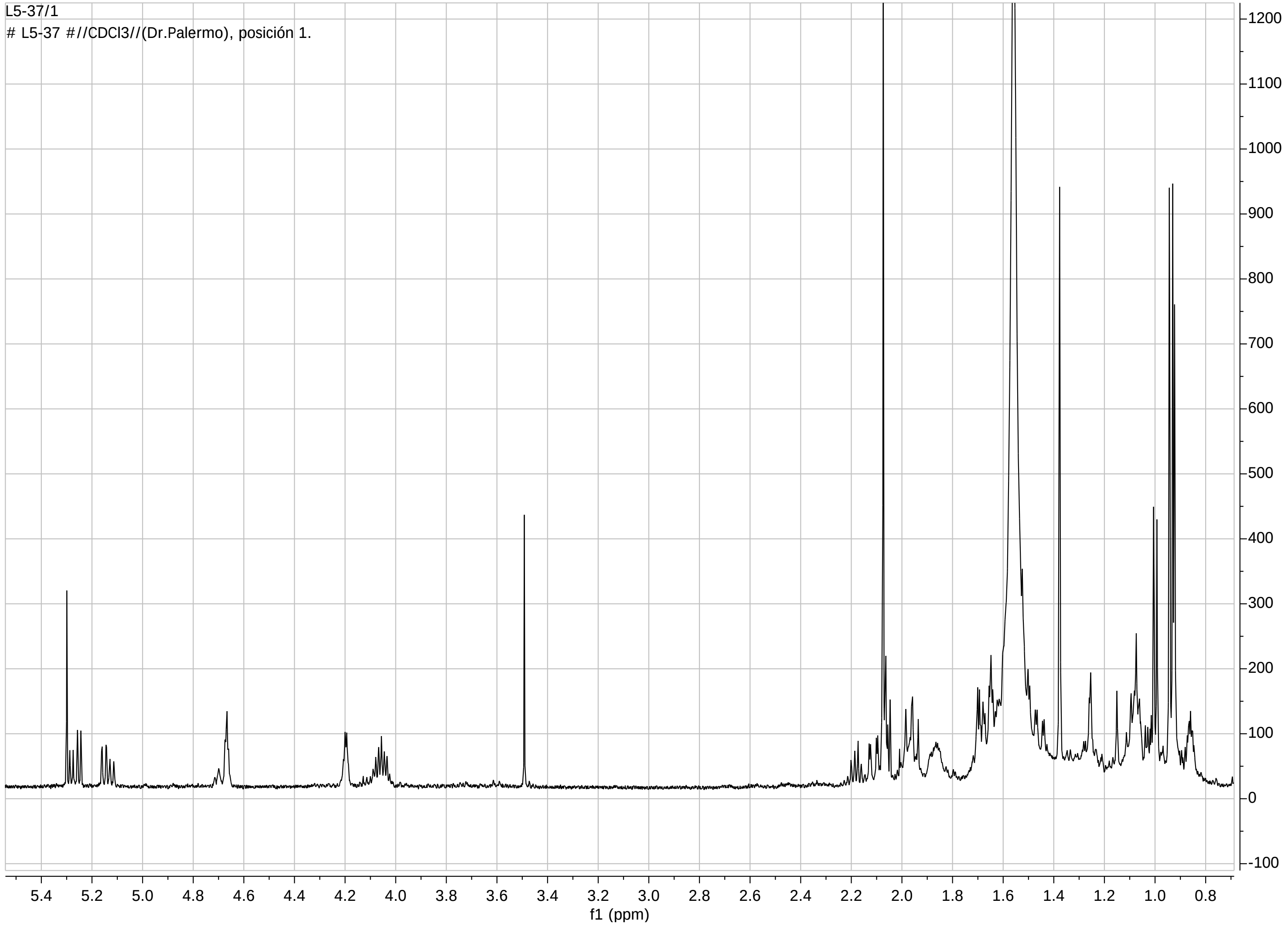

Figure S20. ^{13}C NMR (125 MHz, $CDCl_3$) spectrum of punicinol D (**4**).

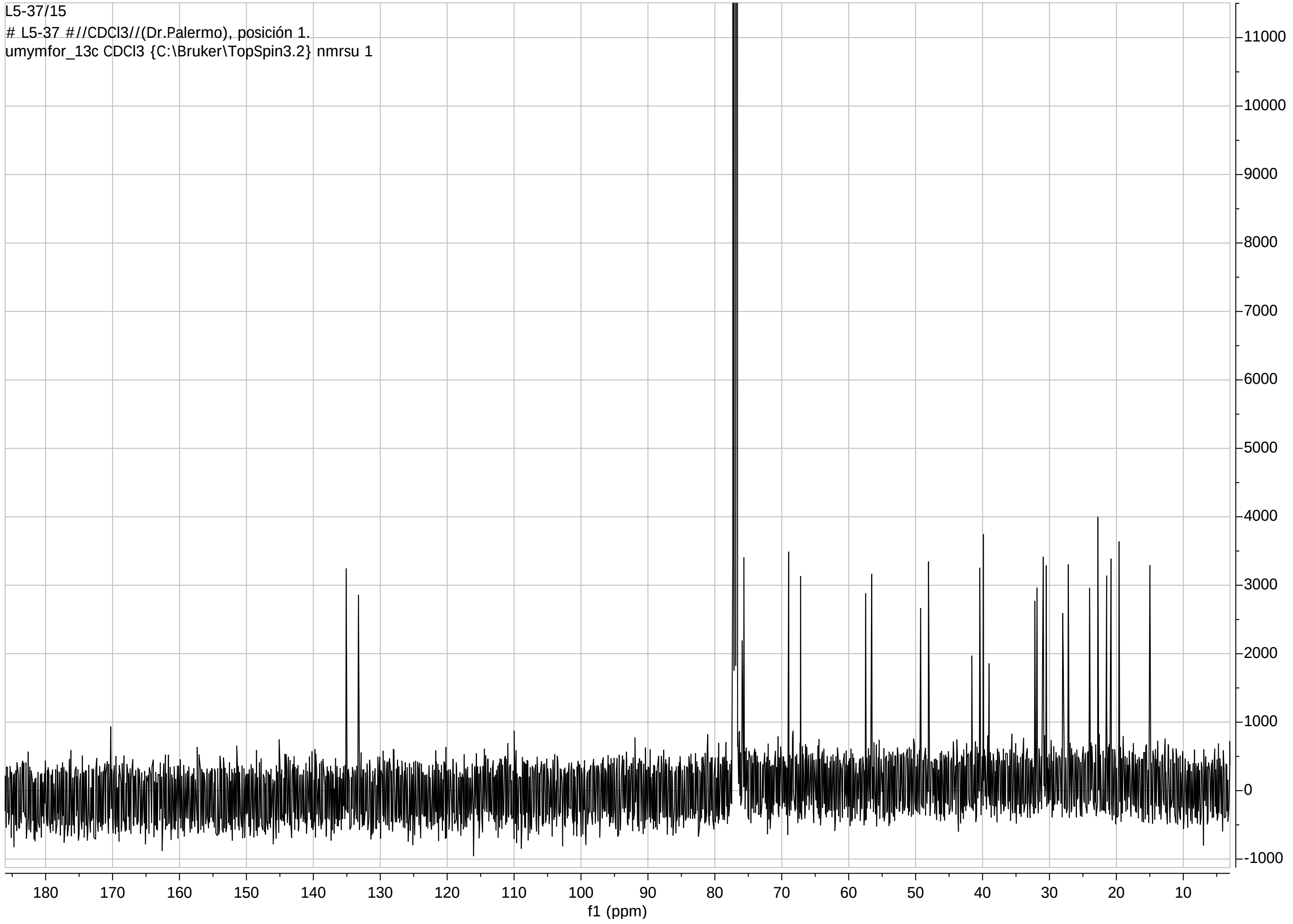

Figure S21. HSQC-DEPT spectrum of punicinol D (**4**).

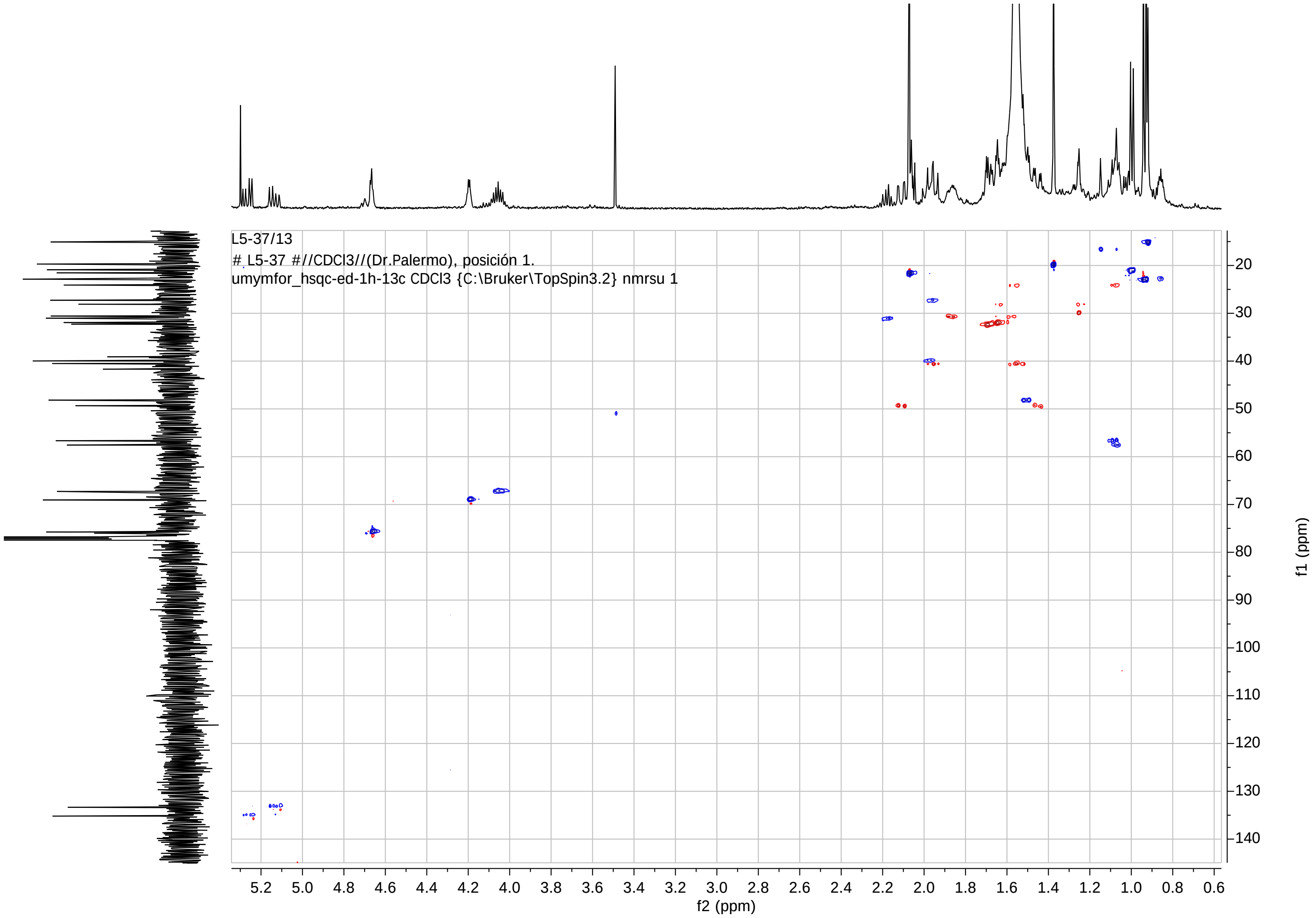

Figure S22. ^{1}H-^{1}H COSY spectrum of punicinol D (**4**).

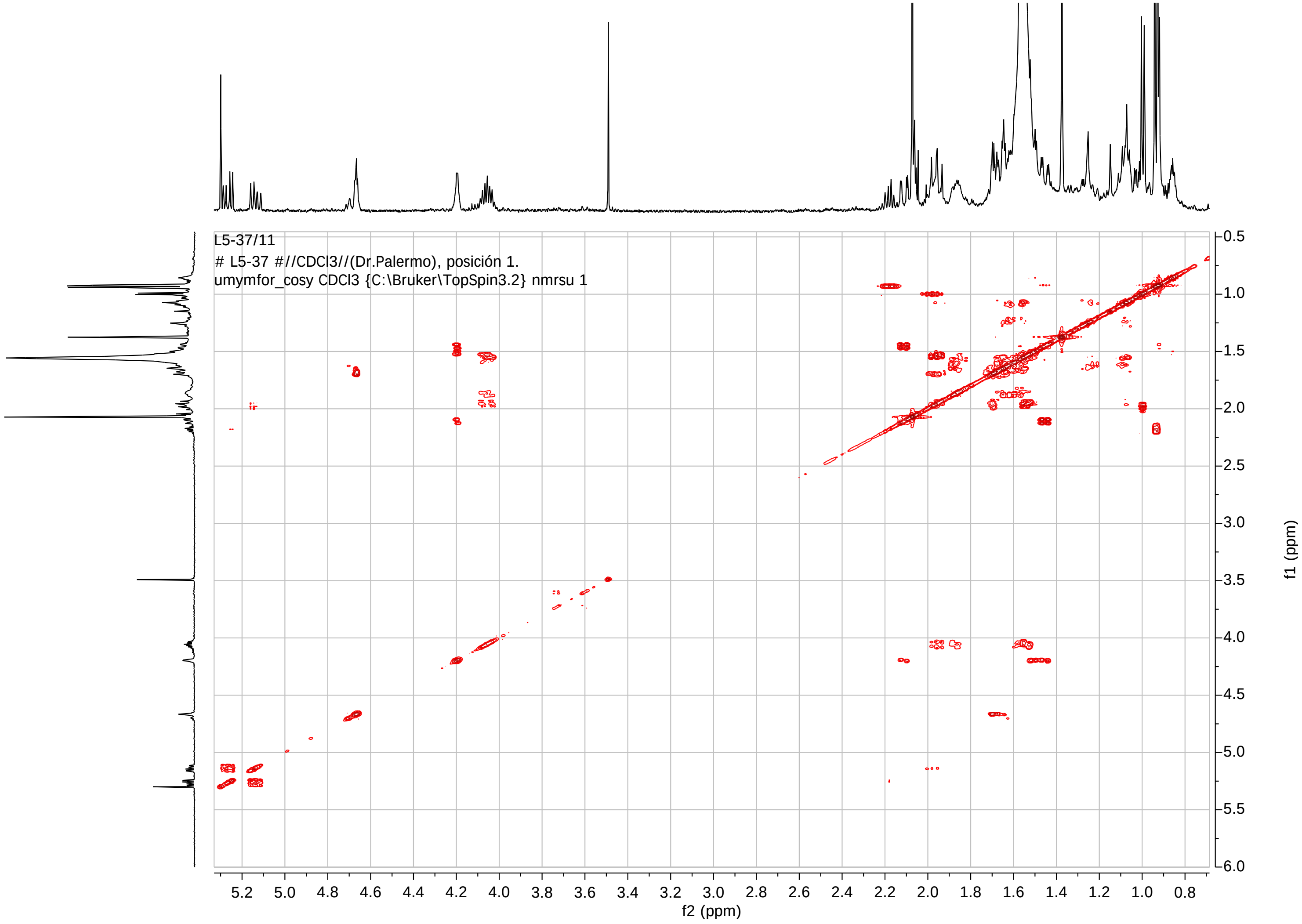

Figure S23. HMBC spectrum of punicinol D (**4**).

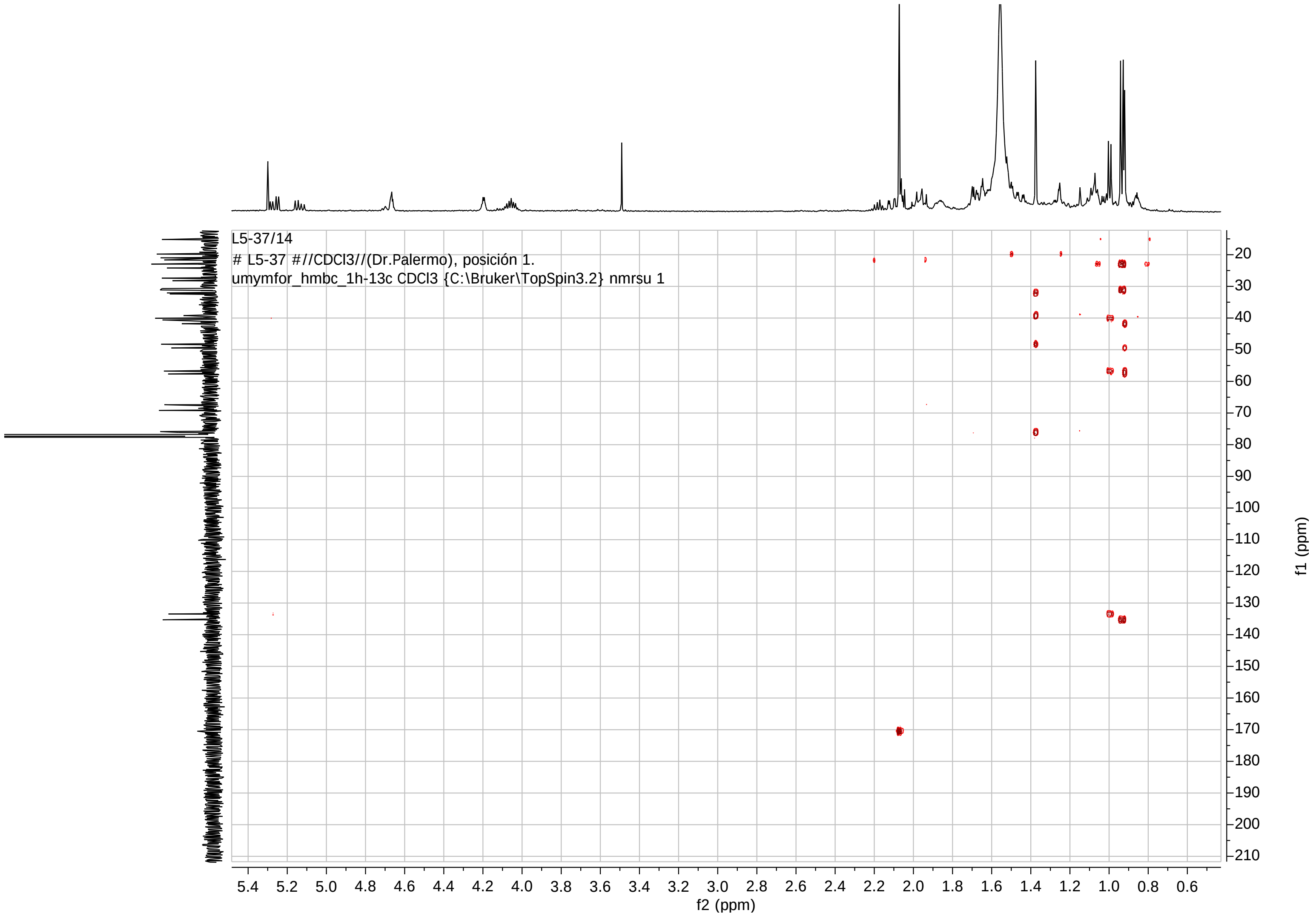

Figure S24. NOESY spectrum of punicinol D (**4**).

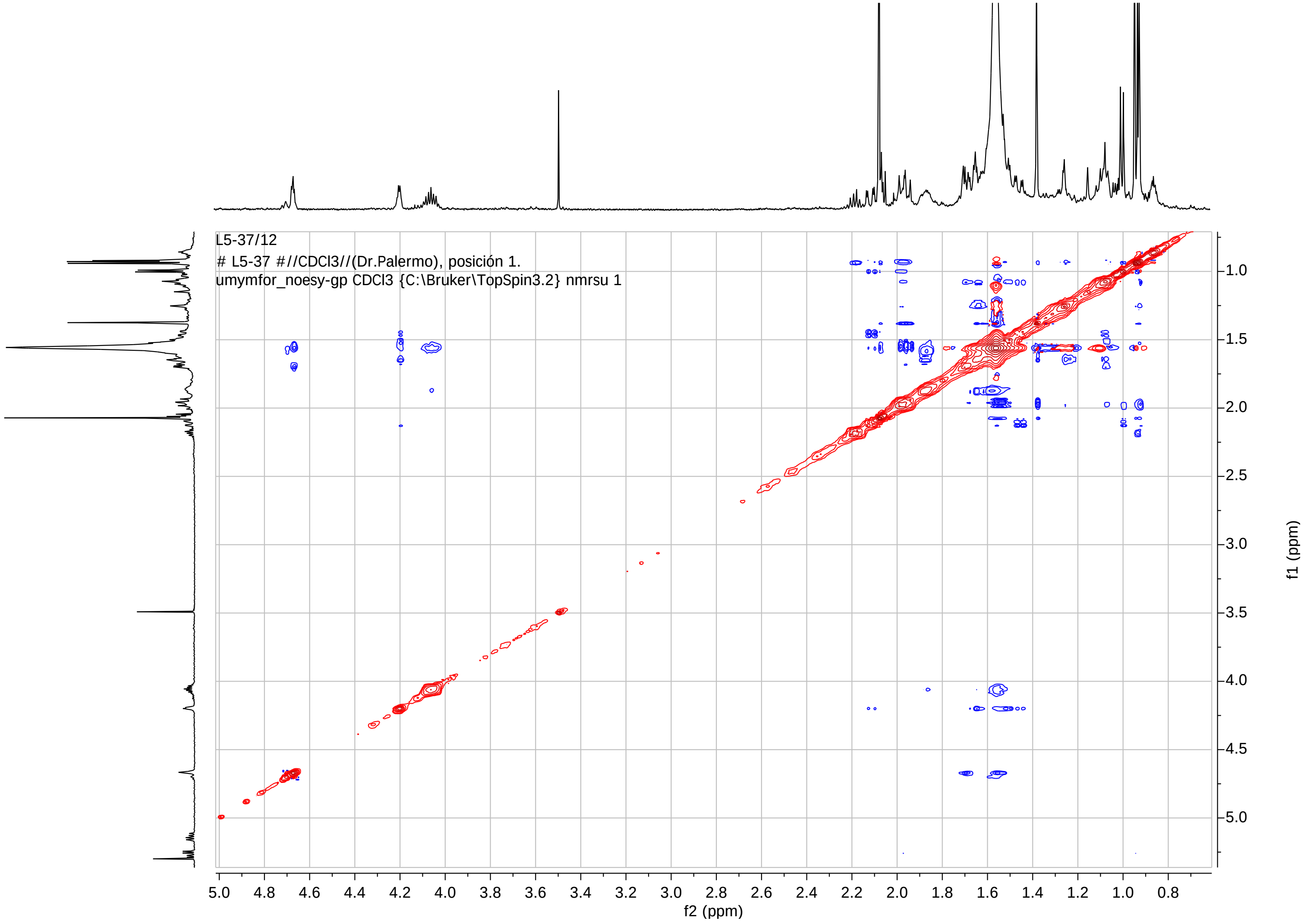

Figure S25. ^{1}H NMR (500 MHz, $CDCl_3$) spectrum of punicinol E (**5**).

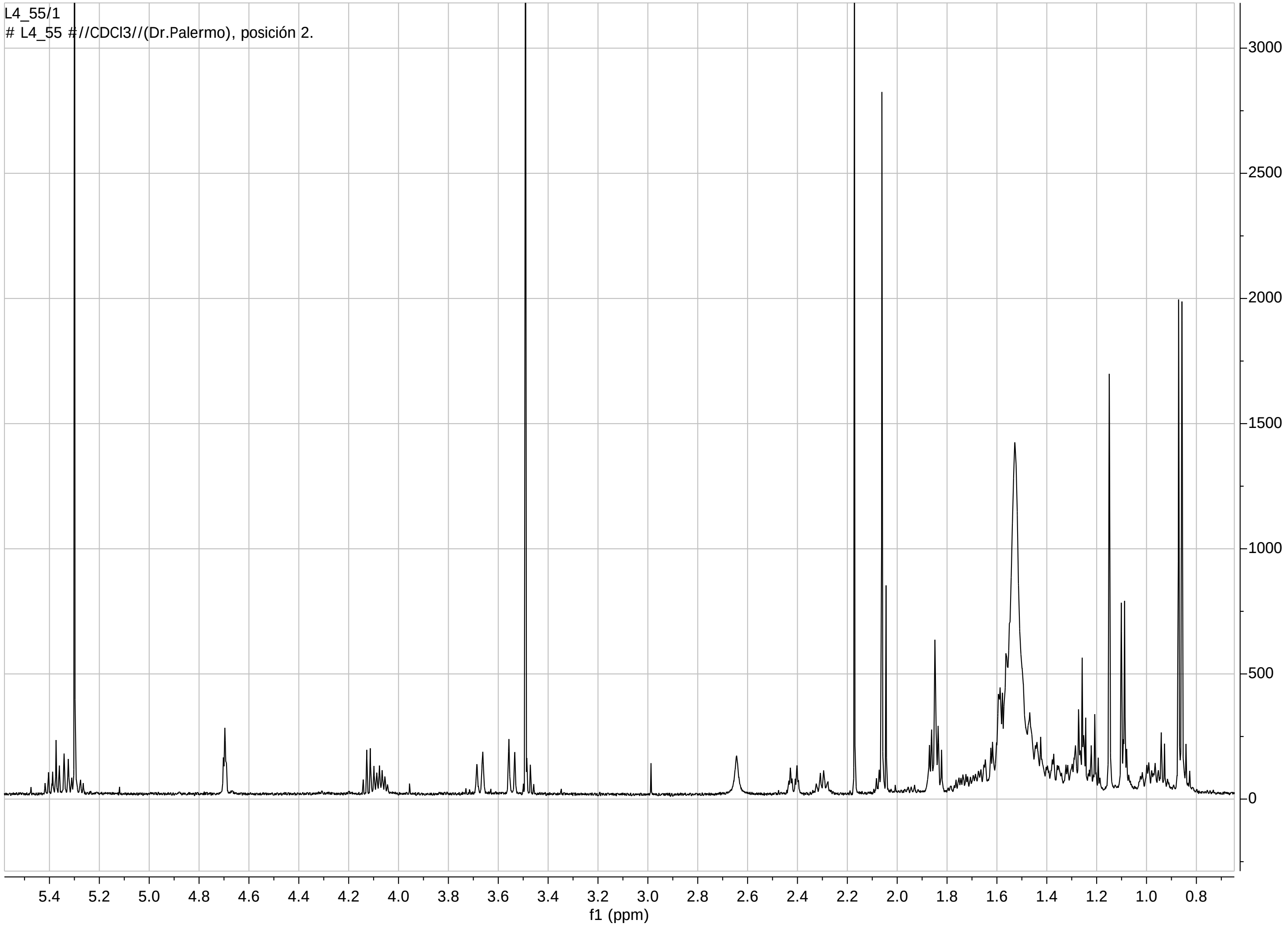

Figure S26. ^{13}C NMR (125 MHz, $CDCl_3$) spectrum of punicinol E (**5**).

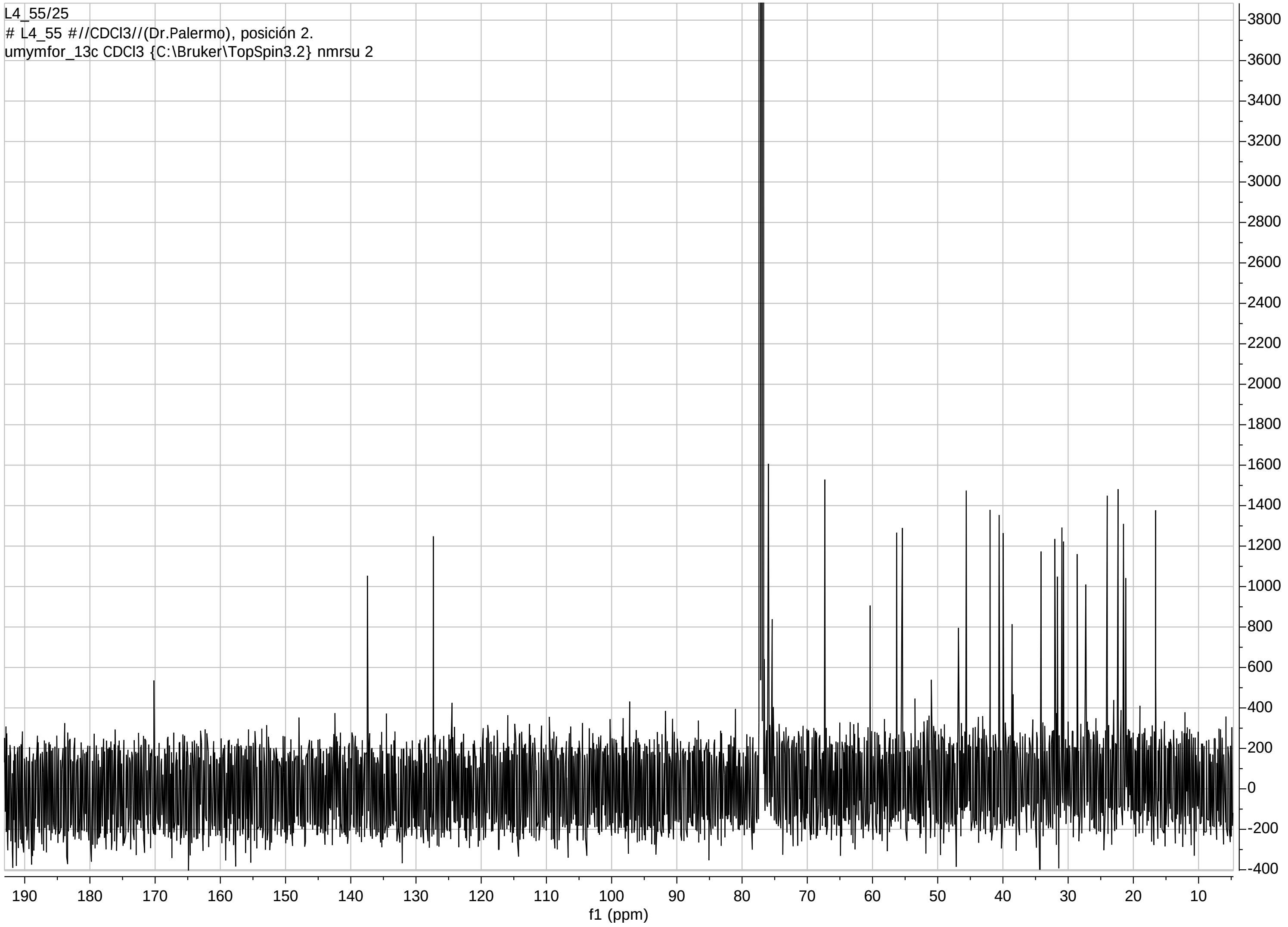

Figure S27. HSQC-DEPT spectrum of punicinol E (**5**).

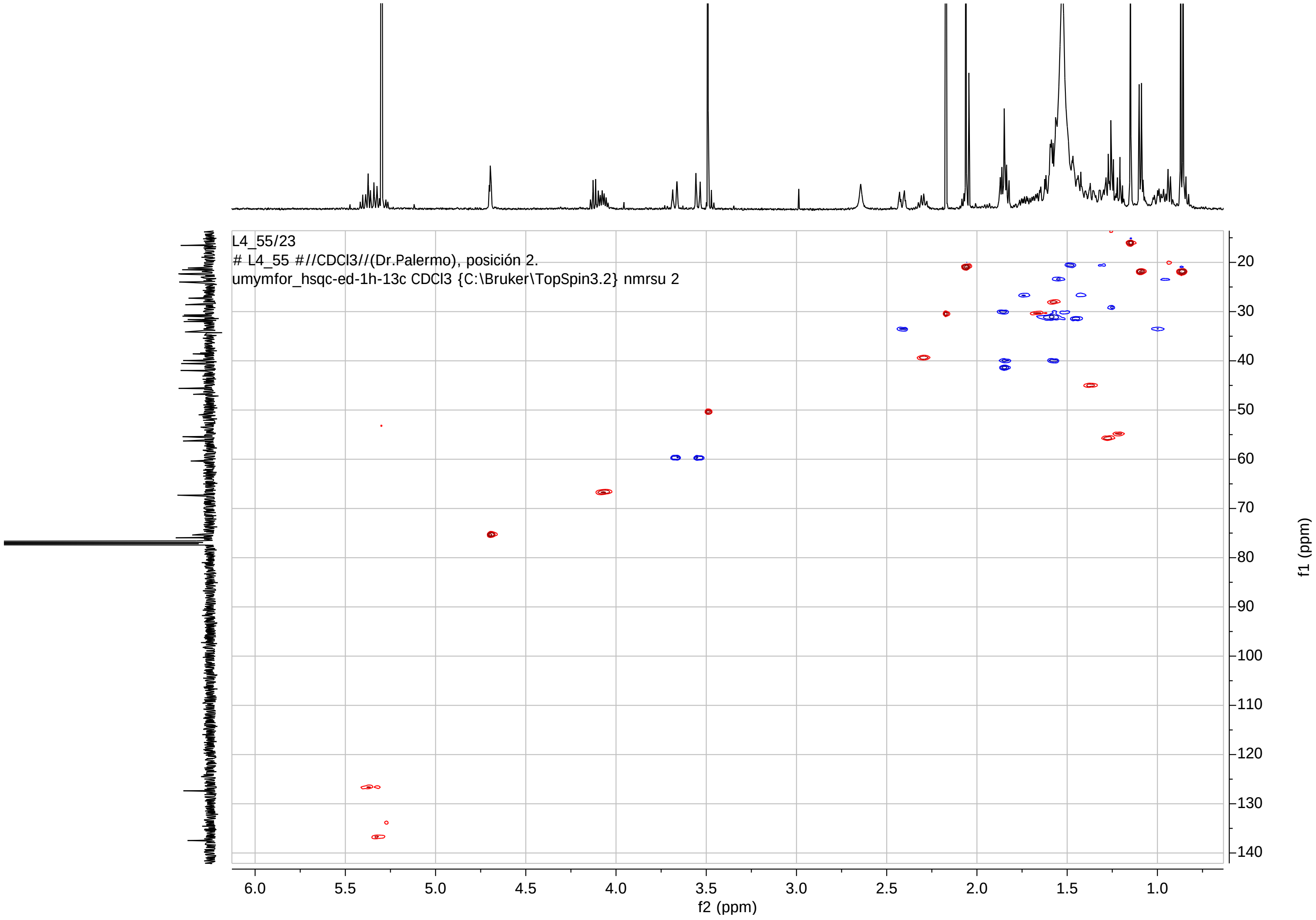

Figure S28. ^1H-^1H COSY spectrum of punicinol E (**5**).

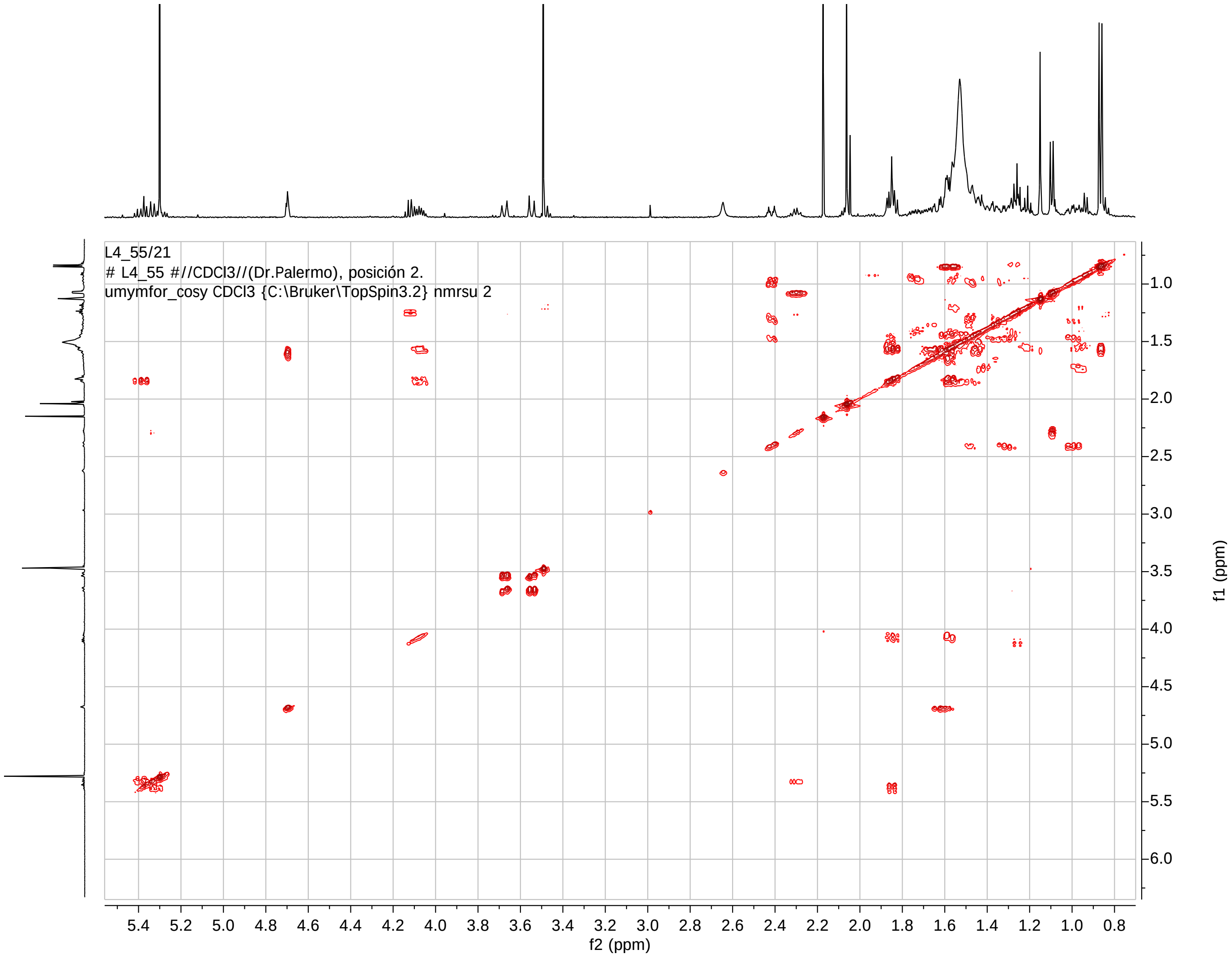

Figure S29. HMBC spectrum of punicinol E (**5**).

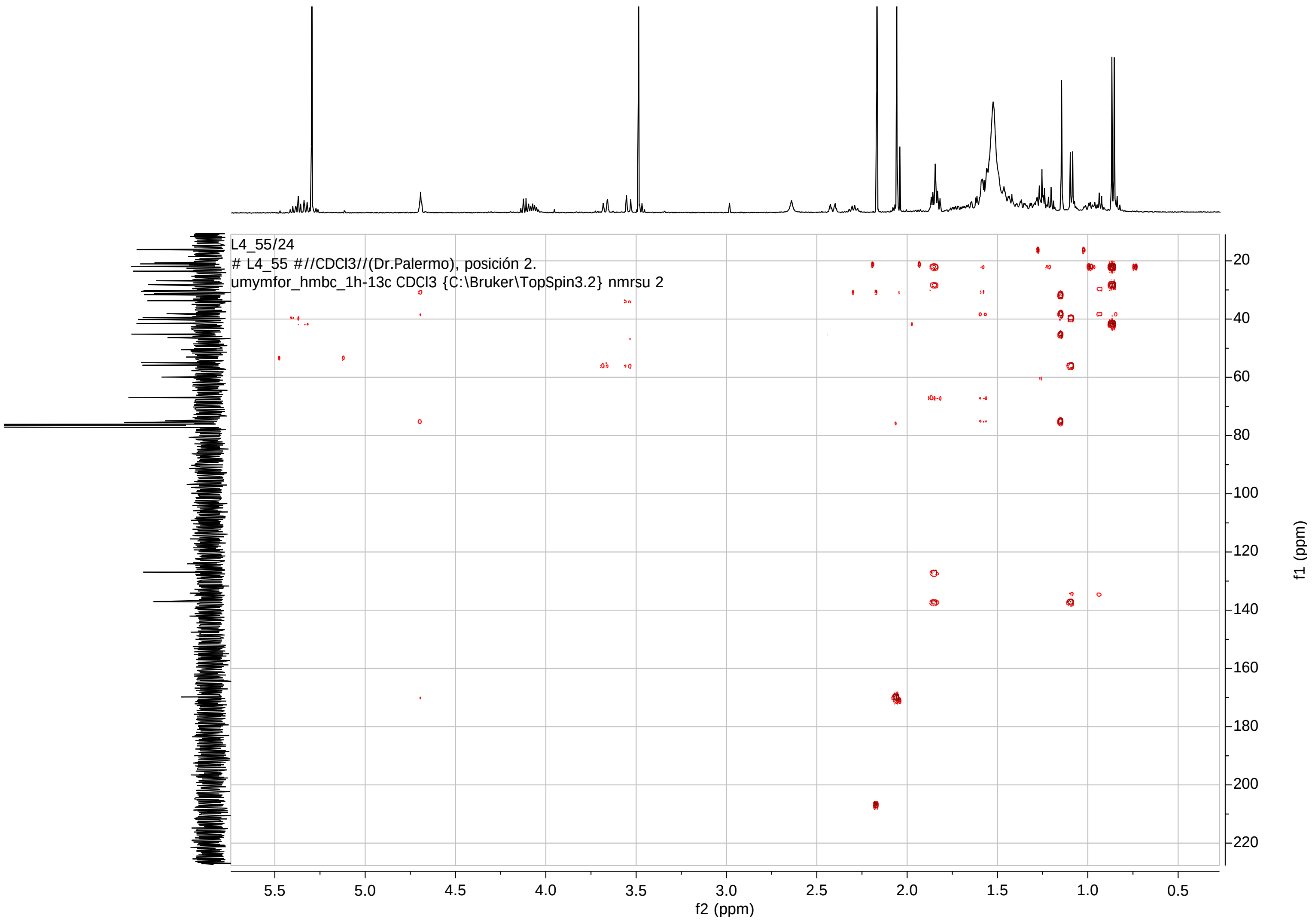

Figure S30. NOESY spectrum of punicinol E (**5**).

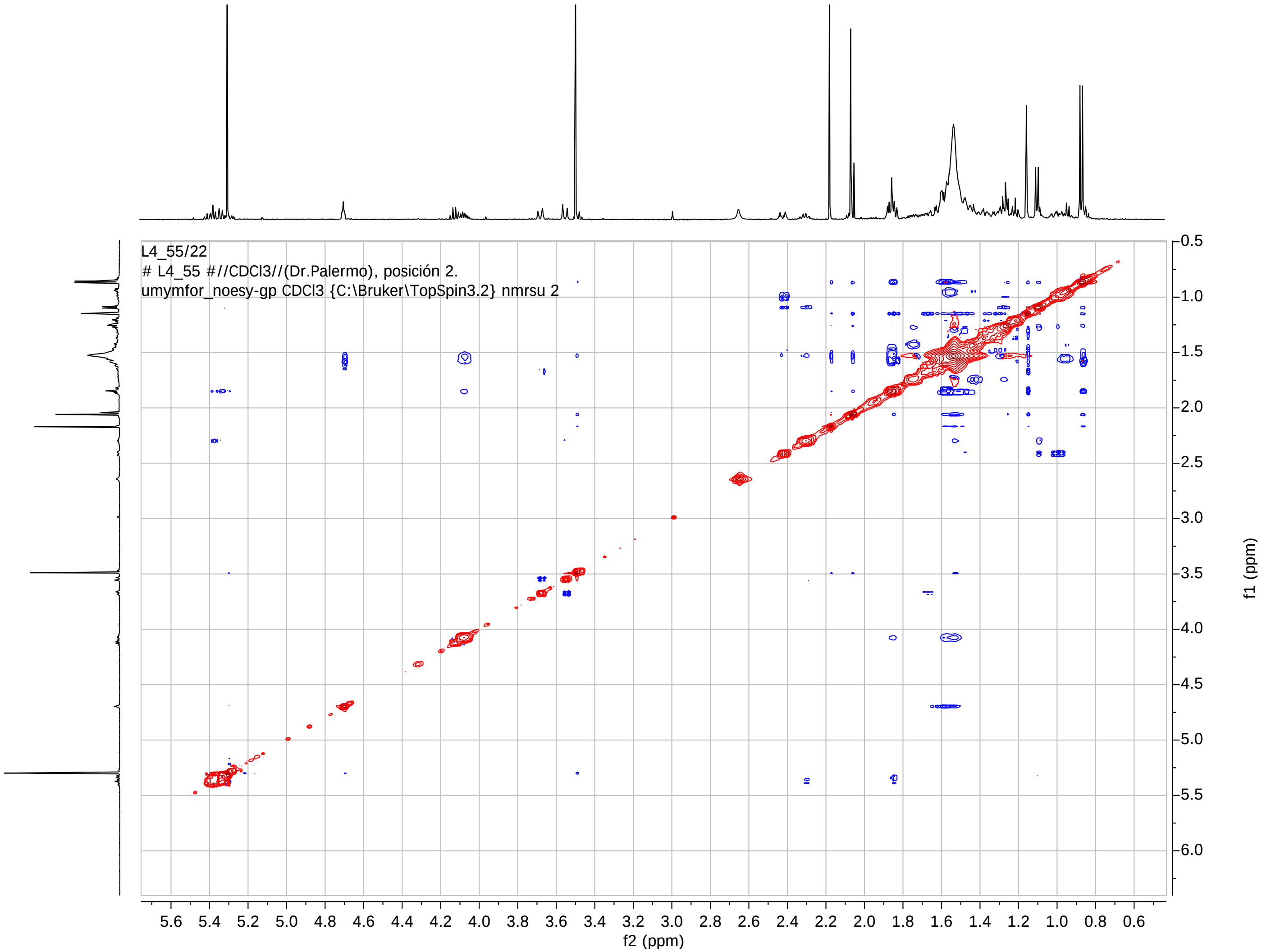

© 2014 by the authors; licensee MDPI, Basel, Switzerland. This article is an open access article distributed under the terms and conditions of the Creative Commons Attribution license (http://creativecommons.org/licenses/by/3.0/).
